# Tools for Decoding Ubiquitin Signaling in DNA Repair

**DOI:** 10.3389/fcell.2021.760226

**Published:** 2021-12-07

**Authors:** Benjamin Foster, Martin Attwood, Ian Gibbs-Seymour

**Affiliations:** Department of Biochemistry, University of Oxford, Oxford, United Kingdom

**Keywords:** DNA damage, DNA repair, genome stability, ubiquitin, CRISPR-Cas9 screen, cryo-EM, cross-linking mass spectrometry, proteomics

## Abstract

The maintenance of genome stability requires dedicated DNA repair processes and pathways that are essential for the faithful duplication and propagation of chromosomes. These DNA repair mechanisms counteract the potentially deleterious impact of the frequent genotoxic challenges faced by cells from both exogenous and endogenous agents. Intrinsic to these mechanisms, cells have an arsenal of protein factors that can be utilised to promote repair processes in response to DNA lesions. Orchestration of the protein factors within the various cellular DNA repair pathways is performed, in part, by post-translational modifications, such as phosphorylation, ubiquitin, SUMO and other ubiquitin-like modifiers (UBLs). In this review, we firstly explore recent advances in the tools for identifying factors involved in both DNA repair and ubiquitin signaling pathways. We then expand on this by evaluating the growing repertoire of proteomic, biochemical and structural techniques available to further understand the mechanistic basis by which these complex modifications regulate DNA repair. Together, we provide a snapshot of the range of methods now available to investigate and decode how ubiquitin signaling can promote DNA repair and maintain genome stability in mammalian cells.

## Introduction

Maintenance of genome stability is critically important for cellular fitness and organismal survival. As such, the genome has to be safeguarded by numerous DNA repair pathways, which are collectively termed the DNA damage response (DDR) (Ciccia and Elledge, 2010). Importantly, defects within the DDR lead to various cancers and contribute to the etiology of various chromosomal instability disorders, so understanding the mechanistic basis of DNA repair is of fundamental importance (Hanahan and Weinberg, 2011; Schumacher et al., 2021). At a broad level, the DDR may be viewed as a highly inter-related signal transduction pathway constructed of DNA lesion-specific sensors, transducers, mediators, and effectors, involving both protein and RNA signaling mechanisms (Jackson and Bartek, 2009). The DDR is also integrated within numerous other cellular pathways, such as the cell cycle and the innate immune response, which allows it to dictate cell fate outcomes after DNA damage (Reislander et al., 2020). Recently, our understanding of these two general principles of DDR function, the inter-relatedness of the DNA repair pathways and integration within other cellular pathways, have been brought into focus as they offer great potential to be exploited for therapeutic purposes (Setton et al., 2021).

Orchestration of the DDR signaling network is performed, in part, by post-translational modifications (PTMs), such as ADP-ribosylation, phosphorylation, ubiquitination and SUMOylation (Dantuma and van Attikum, 2016; Blackford and Jackson, 2017; Palazzo and Ahel, 2018). Protein ubiquitination as part of the ubiquitin-proteasome system (UPS) is carried out by an enzymatic cascade involving E1 ubiquitin-activating enzymes, E2 ubiquitin conjugating enzymes and E3 ubiquitin ligases (Oh et al., 2018). Ubiquitination of protein targets, either by single addition of the ubiquitin molecule or by formation of polymeric ubiquitin chains, provides a multifaceted signaling mechanism to control various aspects of protein function, including localisation, half-life, activation and repression. Ubiquitin signaling is regulated by deubiquitinating enzymes (DUBs), which function to catalyse the removal or trimming of ubiquitin from substrates (Mevissen and Komander, 2017; Clague et al., 2019). In this manner, ubiquitination is a reversible and highly dynamic process within cells, the vast complexities of which we are only beginning to understand (Yau and Rape, 2016). Moreover, given its wide-ranging role in regulating myriad cellular pathways, the ubiquitin system has become a prominent target for drug discovery to treat a range of different pathologies (Rape, 2018; Wu et al., 2020; Duan and Pagano, 2021; Morgan and Crawford, 2021; Tokheim et al., 2021).

The ability of ubiquitin to act in a multitude of processes is largely due to the diverse ubiquitin structures that are formed and then recognised by effector proteins (Swatek and Komander, 2016). The seven internal lysine residues within ubiquitin and the N-terminal methionine can function in polyubiquitin chain formation providing a broad repertoire of ubiquitin chain architectures. Moreover, the existence of heterotypic ubiquitin chains, which can be classified as mixed or branched chain types, further expands the complexity of ubiquitin signaling (French et al., 2021). Ubiquitin can also be modified by ubiquitin-like proteins (UBLs), such as SUMO, or post-translational modifications, including phosphorylation and acetylation (Guzzo and Matunis, 2013; Hendriks and Vertegaal, 2016; Swatek and Komander, 2016; Song and Luo, 2019). Thus, these different mechanisms generate an essentially unlimited number of combinations of ubiquitin chain architectures, referred to as the ‘ubiquitin code’ (Komander and Rape, 2012). In order to decode this signaling, cellular proteins use a range of ubiquitin binding domains (UBDs) to non-covalently interact with ubiquitin, thereby facilitating the transfer of information from the substrate linked ubiquitin chain architecture to the effector protein containing the UBD (Dikic et al., 2009; Mattern et al., 2019). There are at least 20 distinct UBDs in the human genome, many of which display remarkable specificity for ubiquitin chain linkage types and lengths, though they generally exhibit low affinities for ubiquitin (Husnjak and Dikic, 2012). Multiple mechanisms exist to increase avidity between UBDs and ubiquitin, such as combinations of UBDs within the same protein or protein complex, which may help overcome the low affinities of individual UBDs for ubiquitin in cells (Rahighi and Dikic, 2012). It’s possible that the low affinities of UBDs for ubiquitin has prohibited the discovery of a larger repertoire of UBDs in the human genome, with the disconnect between known UBDs and the complexity of the ubiquitin code described as the ‘dark matter’ of the UPS (Radley et al., 2019).

The complexity of ubiquitin chain architectures poses a technical challenge if we are to understand how this ubiquitin code promotes various cellular processes and how its dysregulation impacts disease. To address this challenge, a number of recent methodological approaches have been developed and utilised to better understand the assembly, structure and cellular function of the ubiquitin code. Given that the role of ubiquitination in the DDR has emerged as a key paradigm in understanding genome stability pathways over the last two decades, these new approaches can provide further insight into the mechanisms of the DDR (Jackson and Durocher, 2013; Garcia-Rodriguez et al., 2016). In this review we discuss a number of recent discoveries in the DDR through the lens of ubiquitin signaling, whilst also pinpointing discoveries in each field that could be used at the intersection of the two. We highlight the methodologies used to make these discoveries, potential limitations, and how these tools can be evolved and used to answer remaining questions. To do this we focus on discoveries in the three broad areas of genetics, proteomics and biochemistry, which have helped illuminate our fundamental understanding of DDR mechanisms and the role that ubiquitin plays within them, as well as how this understanding can be harnessed for therapeutic purposes.

## Genetic Approaches to Understanding the DNA Damage Response and Ubiquitin Signaling

### Lessons From RNAi Screens

A genetic screen is a powerful tool with which to ascertain gene function in complex biological signaling networks in both unperturbed conditions and in response to stimuli that engage specific cellular pathways, for example, after exogenous addition of DNA damaging agents. Genetic screens can uncover relationships between genes by comparative analysis of wild type and engineered, typically knockout (KO), cells (a synthetic lethal (SL) screen) or by use of a small molecule inhibitor against a desired protein target of choice in a particular genotypic background (a chemogenetic screen). At the beginning of this century, large-scale genetic approaches were mainly used in tractable model systems such as bacteria, flies, yeast, or zebrafish. However, the leveraging of RNAi technologies into genome-wide tools revolutionised mammalian genetic approaches in the mid-2000s (Berns et al., 2004; Paddison et al., 2004; Silva et al., 2005; Root et al., 2006). For the first time, this new technology allowed targeted large-scale loss-of-function screens in mammalian cells in both forward and reverse genetic approaches, which put it in stark contrast to random mutagenesis approaches or time-consuming low-throughput mouse knockout generation. Practically, genome-wide libraries of short interfering RNA (siRNA) or short hairpin RNA (shRNA) were constructed and used on a per well basis (for shRNA and siRNA) or used in a pooled format (shRNA only), whereby all the shRNA expressing lentiviral vectors are combined in one pool, with one shRNA sequence per vector. The shRNA sequence is linked to a DNA barcode which then allows it to be identified and its abundance quantified in a population of cells by high-throughput DNA sequencing. Typically, viability assays, flow cytometry, or microscopy were used to apply these genome-wide RNAi technologies. However, some major drawbacks to the RNAi-based screening included partial knockdowns and non-specific or off-target effects (Chang et al., 2006; Boutros and Ahringer, 2008). Whilst partial knockdowns may be useful for studying essential genes, the off-target effects require strict awareness of this limitation and requirement for additional validations.

One pertinent example of the off-target effects associated with RNAi came from a screen designed to identify regulators of homologous recombination (HR). Briefly, HR and non-homologous end joining (NHEJ) are the two major DNA repair pathways that respond to double strand breaks (DSBs) in mammalian cells (Scully et al., 2019; Tarsounas and Sung, 2020). Whilst NHEJ promotes the ligation of DSB ends and can operate throughout the cell cycle, HR requires an undamaged donor template from which to perform DNA synthesis, so is active in S/G_2_-phase of the cell cycle. A key step in the HR pathway is the formation of single-stranded DNA (ssDNA), which is first bound by the RPA complex. RPA is then displaced by the RAD51 recombinase via the actions of BRCA1 and BRCA2, allowing RAD51 to perform a homology search (Scully et al., 2019; Tarsounas and Sung, 2020). Understanding regulators of RAD51 localisation at DSBs was therefore an important question to address and formed the basis of a microscopy-based genome-wide RNAi screen (Adamson et al., 2012). However, the authors found that RAD51 is a common off-target hit in siRNA screening, creating many false positives, which was particularly challenging when the screen was designed to identify regulators of HR (Adamson et al., 2012).

Nevertheless, despite these issues, RNAi-based screens have made a major contribution to our understanding of the DDR and its ubiquitin-dependent regulation. For example, a focused DUB-based shRNA screen coupled to immunoblotting led to the identification of USP1 as a key DUB in the Fanconi Anemia (FA) DNA repair pathway (Nijman et al., 2005). The FA pathway senses inter-strand crosslinks (ICLs) in DNA and promotes their unhooking, followed by downstream DNA repair steps (Semlow and Walter, 2021). A key step in the activation of the FA pathway is the monoubiquitination of the FANCD2-FANCI, which can then be reversed by USP1 (Nijman et al., 2005). In addition, an shRNA-based genome-wide dropout screen in response to the ICL-inducing drug mitomycin C identified several new factors in the FA pathway, including the ubiquitin-binding FAN1 nuclease (Smogorzewska et al., 2010).

In addition to these discoveries, genome-wide microscopy-based RNAi screens identified the E3 ligases RNF8 and RNF168 as key components of the ubiquitin-dependent response to DSBs (Kolas et al., 2007; Doil et al., 2009; Stewart et al., 2009). RNF8 and RNF168 promote histone ubiquitination in response to DSBs, helping to recruit two protein complexes, BRCA1/BARD1 and 53BP1-RIF1-REV7. These two complexes antagonise each other at DSBs, and promote either HR or NHEJ, respectively, and therefore represents a decision point for DSB repair pathway choice in cells (Tarsounas and Sung, 2020; Becker et al., 2021).

Lastly, a focused DUB-based siRNA library was used in a number of orthogonal assays for DSB repair phenotypes to establish new roles for members of this enzyme family (Nishi et al., 2014). These are just a small selection of important findings demonstrating how RNAi-based screening approaches helped shape and expand our understanding of ubiquitin-dependent regulation of the DDR. Despite their subsequent loss in popularity over recent years, RNAi-based screening approaches set the foundation for the rapid development of the next generation of eukaryotic functional genomics tools by advancing the platforms, tools and pipelines for genome-wide screening.

### CRISPR-Cas9 Screens

**FIGURE 1:**
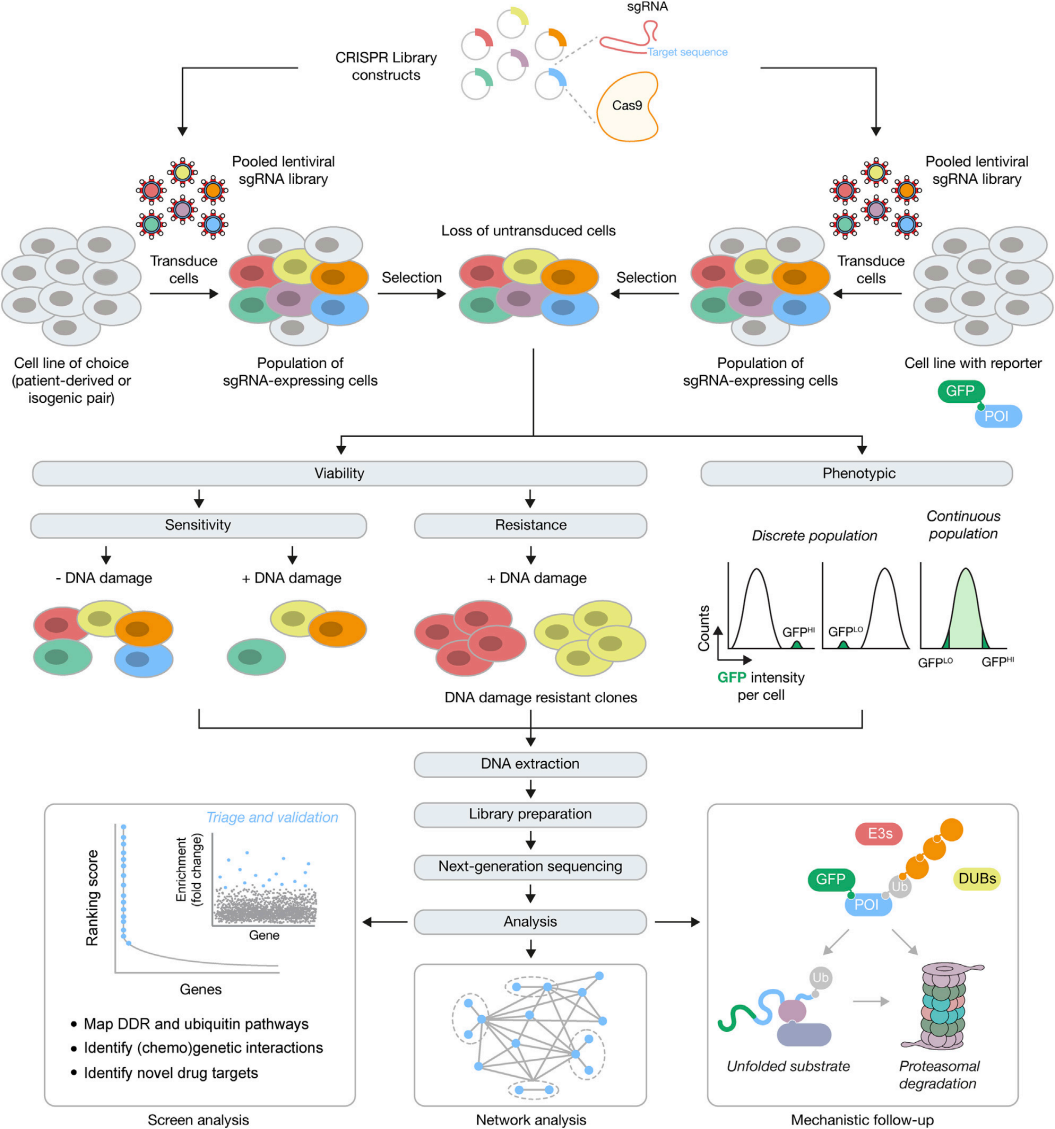
Forward genetics CRISPR-Cas9 screening approaches for the DDR and ubiquitin signaling. *Top*, for genome-wide screens constructs with Cas9 and sgRNAs against every gene are packaged into lentiviral particles, followed by transduction of cells (typically patient-derived, engineered isogenic pairs or cells with integrated fluorescent reporters) at low multiplicity of infection, and then selection for stable integrants. POI, protein of interest. *Middle*, cells with stably integrated constructs expressing Cas9 may now be used in an assay-dependent manner, in viability or phenotypic screens. For viability screens, negative or positive selection can be used to identify genes whose function is essential for survival (e.g., in response to DNA damage) or whose function causes a selective advantage (e.g., resistance in response to DNA damage), respectively. For phenotypic screens, FACS can be used to physically separate the population of cells of choice, depending on the expected population(s) of interest. *Bottom*, after DNA extraction, library preparation and next-generation sequencing, downstream analysis will identify numerous genetic interactors within a DDR or ubiquitin signaling network, that then undergo triage and further validation. After validation, further investigations are needed to understand the mechanistic basis for the genetic interactions. For example, if components of the UPS (E3s, DUBs, protein quality control components and the and proteasome) are found to regulate the GFP-POI, subsequent work will be required to understand the mechanism of this regulation.

Engineering of the bacterial CRISPR-Cas9 system for use in eukaryotic cells led to another leap forward for mammalian functional genomics (Cong et al., 2013; Jinek et al., 2012; Mali et al., 2013). Soon after these ground-breaking discoveries, the CRISPR-Cas9 system was quickly adapted for genome-wide gene essentiality and drug sensitivity/resistance screens in a variety of cancer cell types (Blomen et al., 2015; Hart et al., 2015; Sanjana et al., 2014; Tzelepis et al., 2016; Wang et al., 2015; Wang et al., 2014). Similar to RNAi-based screening, these gene essentiality approaches employ pooled sgRNA libraries in lentiviral vectors, with multiple sgRNAs targeting each gene and each sgRNA sequence linked to a unique DNA barcode, allowing quantification of abundance by high-throughput DNA sequencing (Figure 1). The collective efforts of these monogenic perturbation studies revealed that CRISPR-Cas9 screening identified 3–4 times as many essential genes versus RNAi-based approaches (Hart 2014), with a consensus list emerging of approximately 2000 genes. As expected from RNAi approaches, the UPS ranked highly amongst the essential cellular pathways, as well as checkpoint signaling components of the DDR and components of the DNA replication machinery. However, the majority of human genes can be deleted at no fitness cost to the cell, which in turn presents potential therapeutic opportunities under certain genetic circumstances (Rancati et al., 2018).

The success of CRISPR screening approaches has been underscored by the equitable availability of reagents for performing the screens and the code and software for analysis. However, there are various limitations that need to be considered (Hart et al., 2017; Sanjana, 2017; Doench, 2018). During the assay design of chemogenetic screens, a drug dose is optimised to try to ensure that both sensitisation and resistance effects can be observed in a pooled population of cells for dropout screens. The dose threshold might not reveal more subtle regulators and only identify core nodes of the signaling network of interest. Another limitation is that loss of the sgRNA abundance may reflect some aspect of gene function related to cellular fitness that results in an increased doubling time that, over the time course of the screen, causes the sgRNA to be diluted from the population, but doesn’t mean that gene is essential *per se*. Taking samples at regular intervals during the screen may circumvent this issue. A major potential problem is the extent to which the genome editing causes true KOs or whether the mutants are instead hypomorphs, which could lead to false negatives. In addition, the higher the number of off-target effects, and more DSBs produced, due to copy number variations for example, the greater the likelihood that the lethality phenotype is independent of the on-target gene loss (Meyers et al., 2017). sgRNAs are typically designed to target common exons, however, alternative splicing, in-frame deletions, or inefficient degradation of the mRNA might still lead to protein function. Use of multiple sgRNAs per gene seeks to add statistical robustness in screens, but another approach to ensure KO generation is to target sgRNAs to important functional domains. However, unless the protein is fully characterised then it may have other domains that play different roles in different cellular contexts or pathways and the genome editing may thus just create a separation of function mutant (Shi et al., 2015). Lastly, a potential limitation that hasn’t been fully addressed is whether KO of individual genes cause compensatory regulation of other gene products (Housden et al., 2017). Recent findings from zebrafish found that mutant mRNA production triggered the transcriptional activation of compensatory genes, suggesting that this could be more widespread than fully appreciated (El-Brolosy et al., 2019). Single-cell RNA sequencing coupled to CRISPR-Cas9 editing, such as Perturb-seq (Adamson et al., 2016; Dixit et al., 2016) or CRISP-seq (Jaitin et al., 2016) hold great promise to combine combinatorial genetic perturbations with transcriptomic profiling. Once these approaches are applied at larger scales, perhaps in parallel to single-cell proteomics, they should soon reveal the extent to which genome editing impacts the re-wiring of transcriptional and proteomic profiles.

### Rationale for Targeting the DNA Damage Response

As noted earlier, the DDR is a highly inter-related signaling network, whereby DNA lesions may be channeled from a primary DNA repair pathway to another back-up DNA repair pathway, if the primary DNA repair pathway fails for some reason (e.g., mutation of a particular gene or methylation changes altering gene expression profiles). Thus, in cancers that contain a defect in the primary DNA repair pathway, a back-up DNA repair pathway may repair any DNA lesions that occur, allowing survival of those cancers. However, if the back-up DNA repair pathway is then targeted by small molecule inhibitors, the cancer cell cannot repair the DNA lesions and undergoes apoptosis, effectively targeting and killing the cancer cell. These chemogenetic approaches are therefore a form of synthetic lethality as the inhibition of repair enzymes in the back-up pathways can be viewed as loss-of-function (Setton et al., 2021). This also makes the DDR a highly attractive pathway for identifying therapeutically actionable SL interactions, as healthy cells with both DNA repair pathways can still use the pathway untouched by the small molecule inhibitor, reducing the collateral damage to healthy cells that often occurs in chemotherapies (Lord and Ashworth, 2017). This concept is best illustrated by the identification of a SL interaction between DNA repair enzymes PARP1/PARP2, via small molecule inhibition, and genetic perturbation of the DNA repair genes *BRCA1* and *BRCA2*, which are both mutated in breast and ovarian cancers. BRCA1, an E3 ubiquitin ligase, and BRCA2 function in multiple DNA repair pathways, including HR and fork protection pathways (Tarsounas and Sung, 2020; Qiu et al., 2021; Tye et al., 2021). One major role of BRCA1 and BRCA2 is to facilitate RAD51 loading at DNA lesions, promoting error-free HR DNA repair versus other mutagenic repair pathways. In cancers that contain loss-of-function mutations in *BRCA1* and *BRCA2*, back-up DNA repair pathways exist. One of these pathways is mediated by the PARP family of enzymes, particularly PARP1 and PARP2, which bind to DNA lesions and catalyse the formation ADP-ribosylation signaling to promote DNA repair. Small molecule PARP inhibitors (PARPi) inhibit their ability to produce the ADP-ribose signal, which then traps these enzymes on DNA, as the ADP-ribosylation is also required for their removal from DNA. The PARP trapping causes replication fork collapse upon replisome encounter, which would then require a functional BRCA1/BRCA2 pathway for repair. However, in cancer cells deficient for BRCA1/BRCA2, the lesions resulting from PARP trapping cause irreversible damage that kills the cells (Bryant et al., 2005; Farmer et al., 2005). The seminal findings that inhibition of PARP1/2 in *BRCA1*/*BRCA2* mutated cancer cells selectively kills the cancer cells, but leaves the wild type *BRCA1*/*BRCA2* cells intact, helped define a new epoch in personalised medicine that is already transforming patients’ lives. More recently, the last five years have seen the convergence of newly developed specific DDR drugs and CRISPR-Cas9 screening technologies, the result of which has led to rapid progress in mapping the genetic landscape of the mammalian DDR network and identifying novel SL interactions in the DDR.

### CRISPR-Cas9 Screens and the DNA Damage Response

The success of the PARP-BRCA SL interaction provided the foundation to investigate whether genetic interactions such as this are rare occurrences, or whether there are other SL interactions that are not only therapeutically attractive but provide novel mechanistic insights into the functionality of the DDR network. Furthermore, it led researchers to ask whether there are other genetic determinants that might enhance the PARP-BRCA SL interaction or cause resistance to it, as resistance is a common occurrence in patients treated with PARPi over extended durations. A major breakthrough came after a genome-wide CRISPR-Cas9 chemogenetic viability screen uncovered additional sensitisers to PARPi in three different cell lines (Zimmermann et al., 2018). A high confidence hit across all cell types was RNase H2, an enzyme that functions within the ribonucleotide excision repair pathway (RER) to remove RNA misincorporated into DNA during DNA synthesis. Mechanistically, loss of RNase H2 within the RER pathway causes lesion processing to channel into a TOP1-dependent pathway. These lesions are then recognised by PARP1/2, which are then subsequently trapped at the lesion by PARPi (Zimmermann et al., 2018). An important lesson from this and other papers is that there are numerous ways the efficacy of PARPi-mediated cell death can be enhanced in cells, with PARP1/2 trapping being a focal point that can be increased both genetically and chemically for maximal cell killing effect.

Another major breakthrough in our understanding of the DDR came with the discovery of the Shieldin complex by multiple groups using various approaches (Dev et al., 2018; Ghezraoui et al., 2018; Gupta et al., 2018; Mirman et al., 2018; Noordermeer et al., 2018). Those groups that used genome-wide CRISPR-Cas9 chemogenetic viability screening identified the Shieldin complex with an elegant experimental set-up in which *BRCA1* mutated cancer cell lines or engineered *BRCA1* KO cells, were treated with PARPi at a dose with which the majority of cells are killed (Dev et al., 2018; Noordermeer et al., 2018). However, KO of genes that caused resistance to PARPi would lead to their survival and increased abundance in the population. From here, three previously uncharacterised genes, SHLD1 (C20orf196), SHLD2 (FAM35A), and SHLD3 (CTC-534A2.2), were identified from the CRISPR screens that were then shown to form a complex with REV7, and collectively termed the Shieldin complex. Mechanistically, the Shieldin complex functions downstream of ubiquitin-dependent signaling and the 53BP1-RIF1 axis and binds ssDNA at DSBs via the OB-folds in SHLD2, thereby protecting the DNA ends from BRCA1-mediated resection, RAD51 loading, and engagement of the HR pathway, instead promoting non-homologous end joining (NHEJ) (Dev et al., 2018; Ghezraoui et al., 2018; Gupta et al., 2018; Noordermeer et al., 2018). Use of the NHEJ pathway is more error-prone than HR and in BRCA1-deficient cells treated with PARPi causes lethality through erroneous ligation of broken DNA ends. However, upon loss of the Shieldin complex HR is partially restored in BRCA1-deficient cells, promoting cell survival and resistance to PARPi. The partial restoration of HR in BRCA1- and Shieldin-deficient cells was later shown to be dependent on the RNF168 E3 ligase, which recruits the PALB2-BRCA2 complex to load RAD51 at DNA lesions (Zong et al., 2019; Belotserkovskaya et al., 2020; Callen et al., 2020). In addition, microhomology-mediated end joining via POLQ provides an alternative DNA repair pathway in BRCA1- and Shieldin-deficient cells that is crucial for cell survival (Zatreanu et al., 2021).

Expectedly, much effort has been made to fully explore the PARP-BRCA SL interaction for mechanisms of sensitization and resistance, however, a range of other chemogenetic screens have revealed SL interactions in the DDR (Table 1). A logical culmination to these chemogenetic screening approaches was presented by the Durocher lab, which performed 31 CRISPR screens using 27 different genotoxic agents (Olivieri et al., 2020). Their results provided the first comprehensive genetic map of the DDR in mammalian cells using monogenic perturbation screens, identifying novel components within a network of around 900 genes that cause sensitivity and/or resistance, further underscoring the inter-relatedness of mammalian DNA repair pathways. Given some of the potential limitations to CRISPR screens noted above, it’s likely more factors, especially regulators, of the DDR remain to be uncovered. However, the spectacular progress of this and other chemogenetic screening studies have provided the foundation to move on to combinatorial approaches to dissect gene-gene and gene-gene-drug interactions and beyond.

**TABLE 1:** Selected DDR (chemo)genetic interactions.

Screen type	Assay	Genetic background	Genotoxin	Interactor(s)	References(s)
Resistance	Viability	BRCA1 mutant	PARPi	DYNLL1	He et al. (2018)
Resistance	Viability	BRCA1 mutant	PARPi	Shieldin	Dev et al*.* (2018), Noordermeer et al. (2018)
Sensitisation	Viability	BRCA1 mutant and various	PARPi	RNase H2	Wang et al. (2019), Zimmermann et al. (2018)
Sensitisation	Viability	BRCA1 null	PARPi	CIP2A	Adam et al. (2021)
		BRCA2 null			
Sensitisation	Viability	WT	PARPi	ALC1	Hewitt et al. (2021), Verma et al. (2021)
		BRCA1 mutant			
		BRCA2 mutant			
Sensitisation	Viability	WT	PARPi	HPF1	DeWeirdt et al. (2020), Hewitt et al. (2021)
Sensitisation	Viability	BRCA1 mutant	PARPi	APEX2	Alvarez-Quilon et al. (2020), Mengwasser et al. (2019)
		BRCA2 mutant			
Sensitisation	Viability	Microsatellite instability	-	WRN	Behan et al. (2019), Chan et al. (2019), Lieb et al. (2019)
Sensitisation	Viability	p53	ATRi and MMC	HROB/C17orf53	Hustedt et al. (2019), Wang et al. (2020a)
Sensitisation	Viability	WT	Illudin S and UV	ELOF1	Geijer et al. (2021), van der Weegen et al. (2021)

### CRISPR Screens and Ubiquitin Signaling

Beyond the BRCA1-related pathways above, genome-wide CRISPR-Cas9 screens have uncovered novel mechanisms of other ubiquitin-dependent signaling processes, both within the DDR and beyond, with a few selected examples discussed below. The two most common approaches have involved CRISPR-Cas9 screening in viability assays (sensitivity/resistance) or in combination with flow cytometry. Using viability as an endpoint, several groups used chemogenetic screens to identify gene products whose loss caused resistance to centrosome loss via PLK4 inhibition, including TRIM37 and USP28, and therefore identified components of a centrosome surveillance pathway (Fong et al., 2016; Lambrus et al., 2016; Meitinger et al., 2016). Mechanistic follow-ups revealed that this pathway activates p21-dependent cell cycle arrest after centrosome loss or prolonged mitotic progress via USP28-dependent stabilization of p53. These screens also identified the E3 ligase TRIM37 as a hit that functions independently of the above 53BP1-USP28-p53 pathway, with its loss leading to the formation of centrosome-like structures, thereby causing resistance. This finding was then extended to show that PLK4 inhibition is synthetically lethal with TRIM37 amplification, which is found in 17q23-amplified breast cancers (Meitinger et al., 2020; Yeow et al., 2020). More recently, chemogenetic screens were used to identify regulators of the cellular response to CDK4/6 inhibitors, drugs which are used to treat breast and other cancer types (Chaikovsky et al., 2021; Simoneschi et al., 2021). Both groups identified AMBRA1 as a gene whose loss caused resistance to CDK4/6 inhibition. Further mechanistic investigation revealed that AMBRA1 is part of a Cullin-RING ligase (CRL) four complex, which targets cyclin D for ubiquitination and proteasomal degradation. Thus, in the absence of AMBRA1, cyclin D isn’t degraded, promoting cell and tumour growth that is resistant to CDK4/6 inhibition.

In CRISPR-Cas9 screening approaches that have utilised flow cytometry, fluorescent reporters allow the physical separation of cells exhibiting the phenotype of interest. Typically, the fluorescent reporter is linked to a model substrate or a protein of interest, which allows their abundance to be modulated by genetic perturbation of UPS components. This approach has proved powerful for network mapping and identification of new functions for various components of the UPS, including the E3 ligase RNF185 as a novel regulator of a branch of the ERAD pathway (ER-associated degradation) (van de Weijer et al., 2020), USP5 as a positive regulator of m6A deposition by stabilising METTL3 (Sun et al., 2020), and the ubiquitin-conjugating enzyme/ubiquitin ligase BIRC6 as an autophagy regulator (Jia and Bonifacino, 2019). One limitation to this approach is the potential diversity of substrates for a given pathway. However, evidence shows that it’s possible to detect distinct and overlapping pathways for substrate degradation using multiple model substrates (Leto et al., 2019).

The UPS system of E2s, E3s, and DUBs, is often targeted in genetic screens using smaller, focused libraries. For example, using mammalian cells expressing a fluorescently linked peptidomic library covering the entire human proteome, Koren et al. employed a small scale CRISPR screen to identify adaptors of the CRL families that regulate the rapid turnover of unstable GFP-peptide fusions (Koren et al., 2018). After identification of the adaptors, sequence analysis revealed that the proteins targeted by these adaptors contain a C-terminal glycine residue, thereby providing evidence for proteasomal degradation via a C-terminal degron. Beyond this example, a focused library of E3s and DUBs was used on a large scale to interrogate the UPS for genes that caused sensitivity or resistance to 41 different compounds, each of which target various cellular pathways (Hundley et al., 2021). An interesting observation from this study was that resistance phenotypes could be assigned as being either truly resistant to a compound or that the compound rescued the slow growing phenotype of the genetic alteration. However, this chemogenetic approach was able to assign new mitotic functions to a range of UPS components such as FBXO42, HUWE1, and UBE3D, and will no doubt provide a rich resource for further mechanistic studies. A potential limitation of using focused UPS libraries is that it relies on all the enzyme families being fully annotated and updated with any recent discoveries. Thus, there may be uncharacterised factors that might be missed through focused screening approaches. Moreover, use of only one cell line may limit discovery of important genetic interactions if UPS components exhibit cell-type specific expression profiles, as was recently shown to be the case for the human DUB family (Pinto-Fernandez et al., 2019).

### Genetic Screens and Paralogs Within the Ubiquitin-Proteasome System

Paralogs represent an attractive opportunity to discover new SL targets if the paralogs have maintained some degree of functional relationship. Generally, paralogous genes are less likely to be essential than those genes with no corresponding paralog, suggesting that functional buffering occurs when one of the paralogs is lost, thus paralogs provide ‘genetic robustness’ (Gu et al., 2003; Koonin, 2005). Combined loss of both paralogs may therefore completely ablate function, providing a rationale for pursuing discovery of paralog specific SL targets. Given that several duplication events occurred during the evolution of the ubiquitin system in eukaryotes that led to the generation of numerous paralogous genes (Burroughs et al., 2012; Koonin and Yutin, 2014; Vlasschaert et al., 2017), it will be important to determine the extent of paralog SL and whether this represents a suitable therapeutic opportunity. Encouragingly, evidence beyond the ubiquitin system suggests that this approach might lead to novel and SL interactions as there are numerous pieces of experimental evidence that have revealed paralog SL interactions (Table 2). Furthermore, a computational analysis of over 500 CRISPR screens performed in cancer cell lines suggested that 13–17% of paralog pairs may be SL (De Kegel and Ryan, 2019).

**TABLE 2:** Selected paralog genetic interactions.

Identification method	Genetic background	Paralog #1	Paralog #2	References(s)	
CRISPR knockout screen	HAP1 (chronic myelogenous leukaemia); PC9 (lung adenocarcinoma); A375, MeWo (both melanoma), and RPE-1 (diploid hTERT immortalised)	ASF1A	ASF1B	Kegel et al. (2021), Parrish et al. (2021), Thompson et al. (2021)	
TCGA analysis and hypothesis-driven	HCT 116 (colon cancer); KBM-7 (chronic myelogenous leukaemia) and engineered STAG2 KOs	STAG1	STAG2	Benedetti et al. (2017), van der Lelij et al. (2017), van der Lelij et al. (2020)	
Cancer-dependency dataset analysis and CRISPR knockout screen	Cancers with 18q or 16q loss; PC9 (lung adenocarcinoma)	VPS4A	VPS4B		Neggers et al. (2020), Parrish et al. (2021)
CRISPR knockout screen	786-O (renal cell carcinoma), A375, Meljuso (melanoma), A549 (lung adenocarcinoma), HT-29 (colon cancer), OVCAR8 (ovarian cancer)	AKT1	AKT2/3		Najm et al. (2018)
Cancer-dependency dataset analysis	Chromosome 1p loss	MAGOH	MAGOHB		Viswanathan et al. (2018)
shRNA screen	BRG1-deficient cancer cells	SMARCA2	SMARCA4		Hoffman et al. (2014)
CRISPR knockout screen	A549 (lung adenocarcinoma), HT-29 (colon cancer), OVCAR8 (ovarian cancer); A375, MeWo (both melanoma), and RPE-1 (diploid hTERT immortalised)	FAM50A	FAM50B		Dede et al. (2020), Thompson et al. (2021)
CRISPR knockout screen	A549 (lung adenocarcinoma), HT-29 (colon cancer), OVCAR8 (ovarian cancer); Jiyoye (Burkitt’s lymphoma), K562 (chronic myelogenous leukaemia), KBM-7 (chronic myelogenous leukaemia), Raji (Burkitts Lymphoma)	RPP25	RPP25L		Dede et al. (2020), Wang et al. (2015)

If novel paralog SL interactions are to be discovered in the ubiquitin system, what tools are there to address this? It would be impractical to generate isogenic knockouts of all components of the UPS and perform SL screens in each of them as there are approximately 800 genes. Therefore, combinatorial genetic approaches are required to target at least two loci per cell. Promisingly, a number of these combinatorial approaches have been developed recently, providing scope to address this. The first method, termed CHyMErA, makes use of a Cas9 gRNA and Cas12a (formerly Cpf1) gRNA that are contained within one hybrid gRNA (hgRNA) transcript (Gonatopoulos-Pournatzis et al., 2020). Cells that express both the Cas9 and Cas12a nucleases can cleave the hgRNA due to an inserted Cas12a cleavage site and the RNA cleaving activity of Cas12a. Each gRNA may then direct the corresponding nuclease to its target site. A second approach termed ‘anchor screening’ uses orthogonal Cas9 enzymes from two different bacterial species, *S. pyogenes* and *S. aureus*, to target two different loci in a two-step method (DeWeirdt et al., 2020). In the first step the *S. aureus* anchor sgRNA is delivered to cells together with *S. pyogenes* Cas9. Next, the *S. aureus* Cas9 is delivered together with the library expressing *S. pyogenes* sgRNAs. Thus, only when both steps have occurred is there any genetic perturbation. This approach also negates the necessity for generating single cell clones before carrying out the screen. Lastly, Cas12a has been optimised for pooled screens with multiplexed libraries, with the ability to target up to three genes (Liu et al., 2019; DeWeirdt et al., 2021). Indeed, CRISPR-Cas12a was recently used to screen 400 potential paralog SL interactions using a two-gene approach across 3 cell lines, with 24 SL interactions identified (Dede et al., 2020). Collectively, these combinatorial approaches provide a framework to understand paralog function in mammalian cells. However, when considering paralogs within the UPS, a potential complication is that for most E3 ligases and DUB enzyme classes, there are multiple members, making combinatorial approaches more difficult, but not insurmountable. Specific paralogous pairs to be tested could be stratified and prioritised based on phylogenetic analysis and in combination with various other publicly available datasets.

Interestingly, it’s been shown that ubiquitin paralogs are synthetically lethal in high-grade serous ovarian cancer, as well as other uterine and endometrial cancers (Kedves et al., 2017; McDonald et al., 2017). In mammalian cells, ubiquitin is generated from four genes, *UBB*, *UBC*, *RPS27A* and *UBA5*, with *UBB* and *UBC* producing polyubiquitin gene products. The SL interaction between ubiquitin paralogs arises from the high frequency of *UBB* silencing in these gynecological cancers, causing a dependency on *UBC*, despite the presence of *RPS27A* and *UBA52* (Kedves et al., 2017). This finding therefore identifies the *UBC* gene and mRNA as potential therapeutic targets in these cancer types. However, the mechanistic basis for this SL interaction remains to be determined and warrants further investigation – does the loss of *UBC* exert a global impact on cellular processes through exhaustion of the ubiquitin pools, which the authors termed ‘ubiquitin catastrophe’, or are there other more specific pathways and components whose threshold levels for ubiquitin levels are particularly sensitive and which could be targeted and exploited? Regardless, this reiterates the need for further genetic dissection of the UPS via interrogation of paralog and enzyme class SL interactions.

## Proteomic Approaches for Identifying DNA Damage Response and Ubiquitin Signaling Factors

Whilst the developments of CRISPR-Cas9 screening approaches have provided a powerful tool for mapping genetic interactions in both the DDR and other ubiquitin-dependent pathways, advances in proteomic methods have provided the opportunity to further dissect the protein complexes involved in the DDR, together with how these pathways are orchestrated by ubiquitin signaling. Recently, the development of methods including CHROmatin MASS spectrometry (CHROMASS), nascent chromatin capture (NCC), isolation of proteins on nascent DNA (iPOND), and proximity labelling methods have greatly expanded our understanding of the protein complexes involved in the DDR, and provide the potential to understand how ubiquitin signaling shapes repair events at specific lesions.

### ChEP and CHROMASS

**FIGURE 2:**
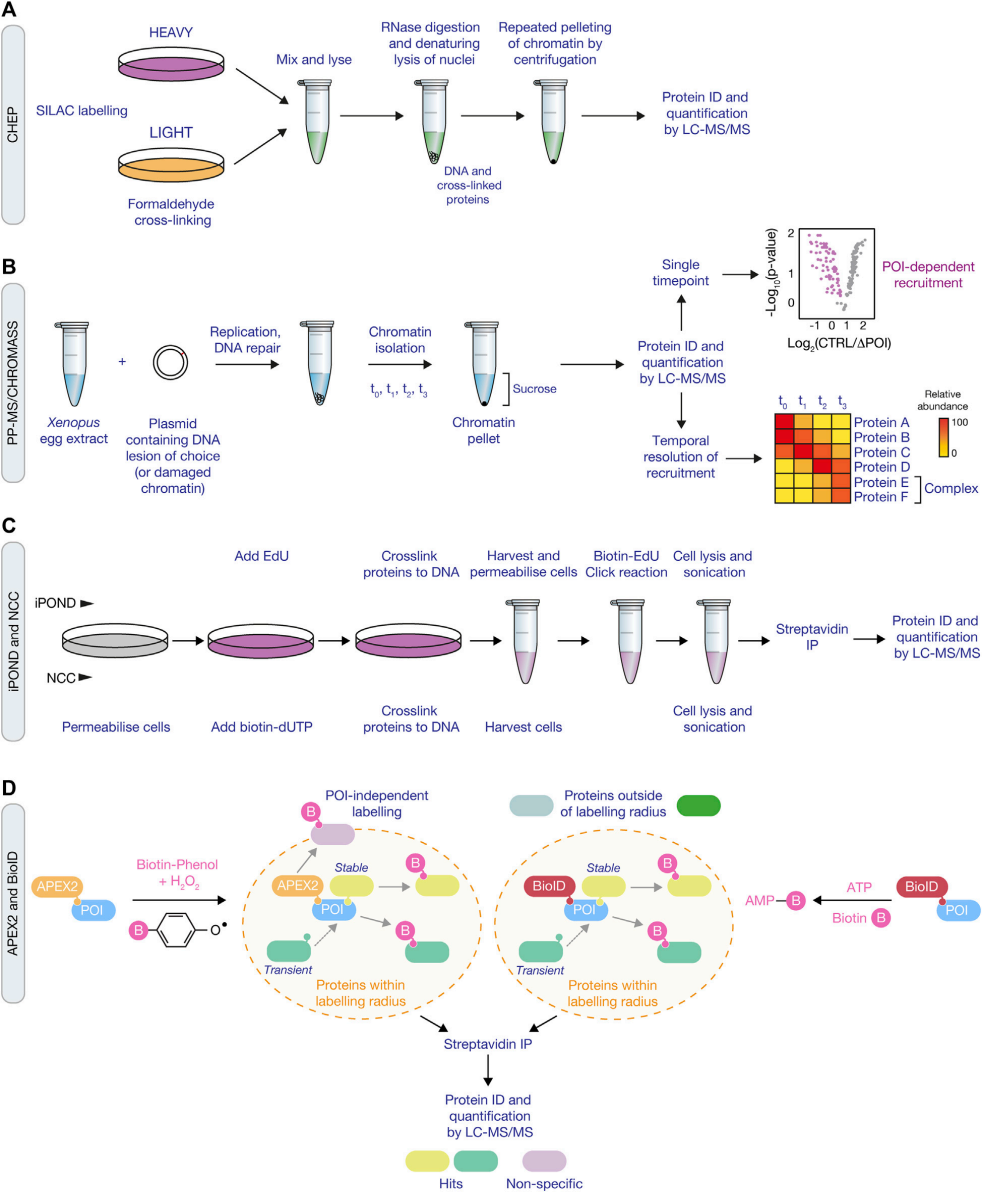
Proteomic approaches for mapping the DDR network and ubiquitin signaling. **(A)** Schematic of the ChEP method, which requires isolation of cross-linked proteins from SILAC-labelled cells to isolate whole chromatin before analysis by LC-MS/MS. **(B)** Overview of the CHROMASS and PP-MS workflows in which damaged chromatin or plasmids with a site-specific DNA lesion, respectively, are incubated with *Xenopus* egg extracts, followed by isolation and subsequent analysis by LC-MS/MS. **(C)** Comparison of iPOND (*top*) and NCC (*bottom*) techniques for identifying factors associated with nascent DNA. iPOND utilises the incorporation of EdU followed by the Click reaction to conjugate biotin to EdU, whilst NCC uses incorporation of biotin-dUTP. **(D)** Schematic of APEX2 **(*left*)** and BioID **(*right*)** proximity labelling methods. APEX2 generates a phenoxyl radical in the presence of biotin-phenol and hydrogen peroxide, resulting in labelling of proximal proteins with biotin. For BioID, biotinylation of proximal proteins uses ATP and is initiated following treatment of cells with biotin to generate biotin-5′AMP to covalently tag proteins. Both approaches use streptavidin pull down to enrich and identify biotyinylated proteins by LC-MS/MS.

A number of approaches have been developed in order to capture and analyse chromatin at a proteomic level, also referred to as the “chromatome” (Kustatscher et al., 2014b). Chromatin enrichment for proteomics (ChEP) is a biochemical procedure to enrich interphase chromatin. ChEP uses formaldehyde cross-linking of chromatin proteins to DNA, followed by isolation of cross-linked proteins by centrifugation under denaturing conditions (Figure 2A). When coupled with mass spectrometry this approach enables analysis of global chromatin composition (Kustatscher et al., 2014a). An integrated chromatin score, based on over 5,000 proteins, defined as chromatin or non-chromatin associated proteins was used to provide a probability score (interphase chromatin probability; ICP) for any given protein to have a chromatin function. ICPs for 7,635 proteins were defined enabling identification of 1840 novel chromatin associated proteins. Whilst ChEP provides a method to globally define chromatin associated proteins, it’s limited by DNA damage that induces the recruitment of a small number of molecules to a lesion, thereby making it difficult to ascertain protein complex recruitment above background levels, or if the protein complex relocalises from one chromatin context to another. Moreover, the method doesn’t allow locus specific enrichment, which is particularly important for understanding protein dynamics within the DDR as repair events invariably occur within discrete foci, for example, at the replication fork or within ionising radiation-induced foci (IRIF).

An alternative method, termed CHROmatin MASS spectrometry (CHROMASS), was developed to identify proteins that are specifically recruited to damaged chromatin (Raschle et al., 2015). This approach utilises DNA replication and repair competent *Xenopus* egg extracts that are incubated with psoralen crosslinked chromatin, followed by chromatin isolation and label-free mass spectrometry to identify proteins bound to the DNA (Figure 2B). To determine recruitment kinetics, chromatin can be isolated at regular intervals to provide a temporal map of the dynamic recruitment of proteins to damaged chromatin. In the first example of its use with chromatin containing psoralen interstrand crosslinks, the authors identified a number of novel DDR factors, including SLF1 and SLF2 (Raschle et al., 2015). Further investigation found that SLF1 and SLF2 form a complex with the ubiquitin E3 ligase RAD18, to promote the ubiquitin-dependent recruitment of the SMC5/6 complex to DNA lesions. CHROMASS has also been used to identify factors recruited to chromatin when DNA replication termination is blocked, including various components of the ubiquitination machinery involved in this process, such as Lrr1 and p97/VCP (Dewar et al., 2017).

The CHROMASS method has since been adapted to investigate protein recruitment dynamics and global analysis of post translational modifications in response to plasmids harboring specific DNA lesions, termed ‘plasmid pull-down with quantitative high-resolution mass spectrometry (PP-MS)’. This approach improves the resolution of the temporal dynamics of protein recruitment versus non-specifically damaged chromatin, and allows for the investigation of specific replication-associated events or lesion-specific DNA repair pathways. As an example, PP-MS was used to map protein recruitment dynamics in response to a defined DNA-protein crosslink (DPC), using a plasmid containing a covalently bound DNA methyltransferase (Larsen et al., 2019). The PP-MS approach identified the SPRTN protease and the recruitment of the proteasome to the DPC, the latter being shown through follow-up studies to be dependent upon TRAIP-dependent polyubiquitination of DPCs, underlining the power of PP-MS as a discovery tool upon which further mechanistic studies can be based (Larsen et al., 2019; Wu et al., 2021). Mechanistic follow-ups are underpinned by the ability to specifically deplete the identified factors from *Xenopus* egg extracts using antibodies. This powerful approach will continue to provide novel insights into various DDR pathways and their regulation by ubiquitin signaling. For example, it may help define the factors and signaling involved in a newly described pathway that repairs acetaldehyde-induced DNA lesions (Hodskinson et al., 2020). Furthermore, as genome editing approaches in *Xenopus* become more robust, this previously genetically intractable system will become amenable to targeted reverse genetics. Further discussion on the *Xenopus* system as a biochemical tool is featured in Section 4 below.

### Nascent Chromatin Capture

Nascent chromatin capture (NCC) was developed to analyse changes in the chromatin proteome of mammalian cells by monitoring biotin-dUTP incorporation of replicating DNA (Alabert et al., 2014). For this approach, cells are released from a single thymidine block and labelled with biotin-dUTP in early/mid-S phase for a short time (5 min) before fixation in formaldehyde to capture the nascent chromatin or chased for 2 h before fixation to capture the mature chromatin. Nuclei are then isolated using a sucrose buffer and chromatin is solubilised by sonication followed by enrichment of biotinylated chromatin by streptavidin beads (Figure 2C). To quantify the composition of nascent and mature chromatin, NCC was combined with stable isotope labelling using amino acids in cell culture (SILAC) to profile 3,995 proteins, providing a comprehensive analysis of proteins enriched in nascent, mature or both nascent and mature chromatin. Moreover, by combining NCC enrichment with a ChEP chromatin probability score, as described above, a chromatin function for 93 uncharacterised proteins was proposed. In an extension to this, NCC-SILAC was recently used to profile protein recruitment in response to different types of DNA replication stress: fork breakage by camptothecin (CPT) or fork stalling by hydroxyurea (HU) (Nakamura et al., 2021)**.** A comparison of the replication fork-associated proteomes identified three classes of replication fork repair factors, with class I and class II factors recruited only in CPT or HU, respectively, and class III factors enriched with both CPT and HU treatment. Class I included DSB (ATM) and HR (CtIP) factors together with PLK1, and class II included factors with known functions in ubiquitin signaling at DSBs, such as RNF168 and RNF169, and the BRCA1-A, FANCI:FANCD2, and SMC5/6 complexes. These findings highlight that NCC-SILAC is capable of detecting distinct fork protein compositions between broken and stalled forks, uncovering novel DDR factors and signaling mechanisms.

### iPOND

Isolation of proteins on nascent DNA (iPOND) is a method that enables purification of newly replicated DNA and its associated proteins from mammalian cells using incorporation and purification of the thymidine analogue 5-ethynyl-2′-deoxyuridine (EdU) (Sirbu et al., 2012). EdU contains an alkyne functional group permitting cycloaddition to a biotin azide by click chemistry, which tethers biotin to the newly synthesised DNA. Following EdU labelling, cells are treated with formaldehyde to cross-link protein–DNA complexes. Cells are then lysed in denaturing buffer and sonicated to fragment the DNA, resulting in solubilised DNA-protein complexes. Biotin enables isolation of DNA-protein complexes using streptavidin affinity purification and detection by immunoblotting or mass spectrometry (Figure 2C). In the first application of this method, iPOND identified a number of replisome components including PCNA and CAF-1 after 2.5 min of EdU labelling (Sirbu et al., 2011). The detection of histones H2B and H3 after 5 min, and linker histone H1 at 20 min following EdU labelling demonstrated that the temporal resolution of iPOND is regulated by EdU incorporation time. Therefore, longer labelling times enable analysis of newly deposited chromatin and assembly, whereas short labelling times capture the components at the replication fork. In a subsequent study, iPOND was used to investigate how replication fork associated proteins are dynamically regulated in response to replication stress. For this, cells were treated with HU or HU and ATR (ATR serine/threonine kinase) inhibitor, to assess how the replisome is impacted by the replication checkpoint (Dungrawala et al., 2015). Proteomic analysis revealed that the collapse of stalled forks which trigger checkpoint activation are distinct from the collapse of forks that start from aberrantly fired origins following inhibition of the replication checkpoint. In addition, novel replisome-associated proteins were identified, including ZNF644 which forms a complex with the G9a/GLP methyltransferase at replication forks. Thus, iPOND is another approach capable of detecting distinct fork protein compositions in response to different DNA lesions, helping to reveal novel factors and their temporal dynamics at the replication fork.

The development of NCC and iPOND methods have both made major contributions to our understanding of the composition of DNA replication forks and mature chromatin in mammalian cells, in both unperturbed conditions and in response to replication stress. However, there are some potential pitfalls that offer avenues for further improvement. Both iPOND and NCC approaches utilise incorporation of a modified DNA base with either EdU for iPOND or biotin-dUTP for NCC. Both of these approaches assume that the modified base is not recognised as DNA damage and assume that the modified base does not affect binding of proteins to the DNA. In longer-term assays, incorporation of either biotin-dUTP or EdU may result in decreased proliferation and increased DDR signaling, indicating that the modified base could impact normal protein recruitment dynamics (Cortez, 2017). The development of native iPOND without formaldehyde cross-linking may circumvent detection issues associated with using formaldehyde and potentially help to provide better access to the labelled DNA by improving efficiency of the click reaction. Moreover, improvements in the efficiency of the click reaction for iPOND and capture of the labelled DNA may help to increase retrieval of proteins at the replisome. Probably the biggest issue with these methods is that even a 10 min pulse of EdU or biotin-dUTP will label, at the very least, approximately 10 kb of DNA in mammalian cells, suggesting that a significant amount of purified DNA will derive from post-replicative DNA. Thus, future approaches might seek to remove as much of the post-replicative DNA as possible. In turn, by increasing the sensitivity and specificity of replisome isolation, it should then be possible to couple such an approach with a secondary purification strategy for PTMs such as ubiquitin, which will not only allow identification of replisome components, but also uncover novel replisome-associated ubiquitin signaling events that have so far remained elusive. Lastly, the recent progress of inducible protein degradation systems for mammalian cells, such as the AID or dTAG systems, provide the tools to deplete DDR and ubiquitin factors in minutes to hours, drawing mammalian approaches closer to the power of the *Xenopus* system when combined with the proteomic approaches described here (Nabet et al., 2018; Yesbolatova et al., 2020).

### APEX2 and BioID

The approaches discussed above rely on stable associations between protein complexes and DNA, which may preclude identification of proteins that only transiently interact with the replisome or are poorly expressed in the cell. As such, proximity labelling may provide an alternative approach to map factors that only transiently interact with the replication fork. APEX, or the more recently developed APEX2, is an engineered peroxidase derived from plant ascorbate peroxidases that can be targeted to a specific subcellular compartment or to a protein of interest. In the presence of biotin-phenol, APEX generates a reactive phenoxyl radical when treated with a pulse of hydrogen peroxide (Figure 2D) (Lam et al., 2015; Hung et al., 2016). This enables the covalent tagging of biotin with nearby nucleophilic electron-rich amino acids such as Tyr (>95%), Trp, His, and Cys of interacting or neighbouring proteins within a small 10–20 nm radius. Biotinylated proteins can then be enriched by streptavidin beads and identified by mass spectrometry. Demonstrating the application of this strategy, Gupta et al. endogenously tagged 53BP1, BRCA1, and MDC1 with APEX2 to generate interaction maps for each of these key DDR factors, which resulted in the identification of the Shieldin complex, the function of which was described above (Gupta et al., 2018).

In addition of the APEX proximity approach, BioID (Biotin IDentification) is a promiscuous mutant of the *E. coli* biotin ligase which can also be used to biotinylate proximal proteins (Roux et al., 2012). In this system, only biotin needs to be supplied to catalyse formation of biotin-5′-AMP anhydride and initiate covalent tagging, preferentially targeting lysine residues (Figure 2D). However, slow kinetics require biotin labelling for 18–24 h to produce sufficient biotinylated material for proteomics. As a result, two variants were identified that could reduce labelling times to 10 min, namely TurboID, a 35 kDa variant with 15 mutations relative to WT BioID, and miniTurbo, a 28 kDa with the N-terminal domain deleted and 13 mutations relative to WT BioID (Branon et al., 2018). Split versions of APEX2 and TurboID have also been developed in which two inactive fragments of the labelling reporters become activated when they physically interact (Han et al., 2019; Cho et al., 2020a; Cho et al., 2020b). Each fragment can be driven together when engineered to detect a specific protein-protein interaction or organelle contact and can provide higher targeted specificity relative to full length enzymes.

Both APEX and BioID strategies are appealing for mapping potential enzyme-substrate interactions involved in ubiquitin signaling, which has proven difficult historically using traditional affinity purification approaches, with a few reports showing promise (Coyaud et al., 2015; Bakos et al., 2018; Dho et al., 2019). However, the use of a 28 or 35 kDa labelling tag may interfere with localisation or function of the bait protein. In addition, bias is generated from the number and accessibility of the targeted amino acid residues of the interacting proteins, and therefore the level of biotinylation does not necessarily correspond to the strength of the association. In addition, the use of hydrogen peroxide at 1 mM for 1 min for proteomic studies to generate the reactive phenoxyl radical by APEX will inactivate DUBs and cause oxidative damage, which could have implications for activation of DNA repair pathways. Despite the improved labelling times (reduced to 10 min) with TurboID and miniTurbo, the reported self-biotinylation of the bait the protein may have some impact and limit accessibility to the full repertoire of interacting proteins (Branon et al., 2018). For both proximity labelling approaches the inclusion of various technical and biological controls, such as cellular spatial references, is essential to determine the specificity of the labelling, as they both suffer from high numbers of false positives from random spatial associations that occur with the bait protein (Lobingier et al., 2017; Go et al., 2021).

## Mass Spectrometry and Chemical Approaches for Decoding Ubiquitin Signaling

As noted above, further improvements in the ability to purify specific structures from mammalian cells in which DNA repair processes are actively being carried out, such as the replisome or IRIF, will pave the way for better sensitivity and specificity of the factors involved and their temporal changes following DNA damage. Furthermore, combining these approaches with the recent advances in ubiquitin mass spectrometry (MS) techniques and chemical biology approaches described below, will be vital for revealing the deep level of DDR regulation by ubiquitin signaling.

### Ubiquitin Site Profiling

Developments in MS methods over the past decade have significantly advanced our understanding of the complex and diverse nature of ubiquitin signaling in cells, as well as the enzymatic machinery responsible (Vere et al., 2020). Prior to these advances the ability to detect ubiquitinated sites relied on expression and enrichment of tagged ubiquitin from cells. For example, expression and enrichment of His-tagged ubiquitin from *S. cerevisiae* allowed MS-based detection of ubiquitinated peptides after identification of the signature di-glycine (K-GG) remnant of ubiquitin, which remains covalently attached to the target lysine after trypsinisation (Peng et al., 2003). In this study, 72 ubiquitinated proteins were identified with 110 ubiquitination sites, including identification of modifications on ubiquitin at lysine residues. Since this study, tagged ubiquitin variants have been used to identify ubiquitinated substrates in mammalian cells, however, they have suffered from an inability to conclusively identify the specific ubiquitination sites, hampering further mechanistic studies from these datasets (Kirkpatrick et al., 2005; Tagwerker et al., 2006; Danielsen et al., 2011; Oshikawa et al., 2012).

**FIGURE 3:**
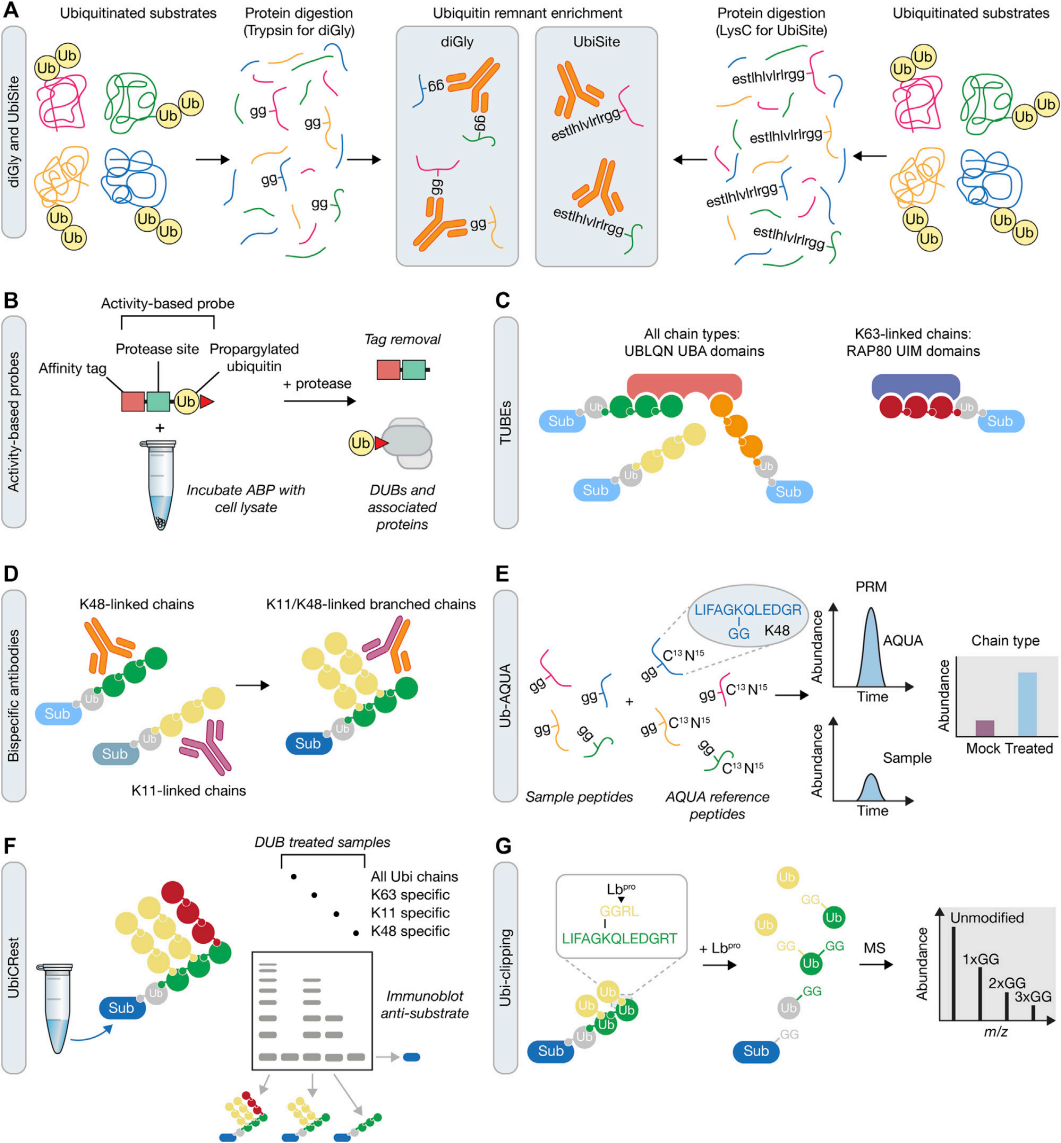
Mass spectrometry approaches for understanding ubiquitin signaling. **(A)** Prior to mass spectrometry, ubiquitin remnants can be enriched following either trypsin or LysC protein digestion. These approaches rely on antibodies that recognise either the di-glycine (diGly) remnant peptide following tryptic digestion of ubiquitin using the K-GG antibody **(*left*)**, or a longer sequence that is recognized by the UbiSite antibody **(*right*)**. **(B)** Activity-based probe (ABP) tools in the UPS. Irreversible reactivity of the ABP with an enzyme’s active site, in this case a DUB, enables purification of the probe-enzyme complex, prior to downstream use, e.g., for mass spectrometry or crystallisation. **(C)** Tandem Ubiquitin Binding Entities (TUBEs) contain a number of specific ubiquitin binding domains that allow for the capture of polyubiquitinated proteins. TUBEs can be designed to capture all ubiquitin chain types (e.g., four UBA domains from UBLQN) or specific ubiquitin chain types (e.g., three UIM domains from RAP80 that bind K63-linked chains). Sub, substrate. **(D)** In contrast to single ubiquitin linkage-specific antibodies **(*left*)**, bispecific antibodies **(*right*)** contain arms from each of these two antibodies that can be used to determine formation of branched ubiquitin chains in downstream assays, such as immunofluorescence, immunoblotting or mass spectrometry. **(E)** Ub-AQUA allows for absolute quantification of ubiquitin linkages of trypsin digested samples that have been spiked with heavy-labelled reference peptides and subsequently detected by multiple reaction monitoring or parallel reaction monitoring. **(F)** UbiCREST uses a panel of linkage-specific DUBs to treat ubiquitinated samples to provide a qualitative gel-based method to assess substrate ubiquitin chain architecture. **(G)** Ubi-clipping uses the Lb^pro^ viral protease to cleave ubiquitin after R74 which, when combined with middle-down MS, provides a quantitative approach for detecting branched ubiquitin linkages.

A major breakthrough for detecting ubiquitination sites came with the generation of an antibody against the resulting K-GG remnant after tryptic digestion of ubiquitin (Figure 3A) (Xu et al., 2010). Utilisation of the K-GG antibody increased the detection of ubiquitinated peptides to 19,000 on approximately 5,000 proteins (Kim et al., 2011). Furthermore, application of this new tool within a DDR context revealed the widespread global extent of DNA damage-driven ubiquitin signaling, as well as leading to novel ubiquitin-dependent repair mechanisms at a single protein level (Povlsen et al., 2012; Elia et al., 2015). Whilst the number of identified ubiquitinated sites is increased by ubiquitin peptide level enrichment relative to protein level enrichment, the K-GG remnant is also present following tryptic digestion of the UBLs NEDD8 and ISG15. In addition, it has been reported that the K-GG antibody has certain amino acid preferences near the modified lysine and also fails to detect N-terminally ubiquitinated proteins (Wagner et al., 2012). In an attempt to circumvent these issues, an antibody was generated that detects the 13 residues at the C-terminus of ubiquitin that remain attached to modified peptides following LysC digestion (Akimov et al., 2018). This approach, termed UbiSite, is specific to ubiquitin and can also detect N-terminal ubiquitination sites. Following sequential LysC and trypsin digestion, UbiSite enabled identification of over 63,000 unique ubiquitination sites on 9,200 proteins in two human cell lines. This approach profiled ubiquitinated proteins of diverse function and localisation and did not show preference for amino acids near the modified lysine, indicating an improved strategy for unbiased identification of ubiquitination sites. Recently, data-independent acquisition (DIA) has been gaining momentum as an alternative approach to extract peptide fragment information from mass spectrometry. DIA continuously acquires both MS1 and MS2 spectra without any bias to precursor ions, unlike data-dependent acquisition (Ludwig et al., 2018). Use of DIA in combination with the K-GG antibody has provided yet further depth in precisely and accurately quantifying ubiquitination sites, highlighting its use as a major future tool in understanding ubiquitin signaling in the DDR at unprecedented detail (Hansen et al., 2021), especially when combined with enrichment strategies discussed above.

### ABPs

In order to investigate the ubiquitin conjugation and removal activities within a cell, chemical-based approaches have been developed, referred to as activity-based protein profiling (ABPP) (Hewings et al., 2017). This method utilises activity-based probes (ABPs) that mimic an enzyme’s substrates and which become covalently attached to enzyme active sites. ABPs can therefore help determine enzyme activity, which can be applied to study the biological function of components of the UPS on a global proteome-wide scale (An and Statsyuk, 2016; Byrne et al., 2017; Mulder et al., 2016; Pinto-Fernandez et al., 2019). ABPs generally consist of an epitope tag for isolation of labelled proteins, a recognition substrate such as ubiquitin, and an electrophilic warhead that reacts irreversibly with the catalytic residues of the enzyme (Figure 3B). The development of ABPs with different thiol reactive groups has proved particularly valuable in the profiling of DUB activity. Initial profiling of a DUB using an ABP was performed using ubiquitin vinyl sulfone (UbVS), which identified USP14 as a proteasome-associated DUB (Borodovsky et al., 2001). Subsequent ABP designs have demonstrated that the electrophile used imparts reactivity towards different DUBs (Borodovsky et al., 2002). Propargylated ubiquitin (Ub-Prg) can react with cysteine residues in the DUB active site forming a vinyl thioether linkage and providing a selective cysteine DUB ABP (Ekkebus et al., 2013). The application of ABPs in understanding the DDR is highlighted by the recent identification of ZUP1, the founding member of a novel class of DUBs (Hewings et al., 2018; Kwasna et al., 2018). In these studies, Ub-Prg was incubated with mammalian cell lysates followed by mass spectrometry to identify cysteine-based DUBs. ZUP1 was readily modified by Ub-Prg, with subsequent analysis confirming that it as an active DUB with specificity for cleaving K63-linked polyubiquitin and a function in maintaining genomic stability (Haahr et al., 2018; Hermanns et al., 2018; Hewings et al., 2018; Kwasna et al., 2018). With ABPs against E1 (An and Statsyuk, 2016), E2 (Mulder et al., 2016), E3 (Byrne et al., 2017) and DUB (Hewings et al., 2017) enzymes, there is now an extensive toolkit with which to analyse temporal activity changes in response to DNA damage when coupled to mass spectrometry and sample multiplexing methods, such as SILAC and tandem mass tagging (TMT).

### TUBEs and Bispecific Antibodies

Often the low stoichiometry of ubiquitination on target proteins makes it difficult to detect the ubiquitinated form from cell lysates. As such, there is a requirement for an enrichment step prior to mass spectrometry or other downstream analytical methods, such as immunoblotting. Coupled to this, there is also a need to purify endogenously ubiquitinated proteins, rather than rely on over-expression of ubiquitin. A tool that addressed both these requirements was the development of Tandem Ubiquitin Binding Entities (TUBEs), which are synthetic constructs that contain multiple UBDs. TUBEs were initially based on the tandem repeated ubiquitin associated (UBA) domains from Ubiquilin and HR23A (Hjerpe et al., 2009). The combination of UBA domains increases the affinity for polyubiquitinated proteins which, when combined with an epitope tag, provides an enrichment strategy to purify ubiquitin chains before MS-based methods (see below) (Figure 3C). Design of the TUBEs can be further modified to capture specific polyubiquitin linkages using linkage-specific ubiquitin binding domains, such as the ubiquitin interacting motifs (UIMs) from RAP80 that bind K63-linked ubiquitin chains (Sims et al., 2012; Mattern et al., 2019). A modified form of the TUBE is the trypsin resistant (TR)-TUBE, which can be expressed in cells to prevent the action of DUBs and proteasomal degradation by acting as a ‘molecular shield’ on the polyubiquitinated chains, providing improved characterisation of the numerous ubiquitination events occurring under steady state conditions (Yoshida et al., 2015). Alternatively, recombinant TR-TUBEs can be used to determine the length and composition of ubiquitin chains purified from cell lysates in combination with MS-based approaches (Ub-AQUA-PRM – see below), in a method termed Ub-ProT (Tsuchiya et al., 2018).

A broader range of linkage-specific TUBEs is limited by several challenges. Perhaps most importantly, for the less well studied atypical and heterotypic chain types, there is a paucity of data about the readers of these chain types and hence the UBDs, or combination of UBDs, that could be leveraged in a TUBE. This difficulty is in part also linked to the ability to distinguish between low affinity and indirect binders of different ubiquitin chain topologies. Thus, by closing these knowledge gaps, the available tools should expand concomitantly, allowing the development of a full repertoire of TUBEs that may then be used to explore the biological function of different ubiquitin chain types and architectures.

Over the last few years, there has been a growing understanding of the functional importance of heterotypic ubiquitin chains in multiple cell processes (Haakonsen and Rape, 2019; French et al., 2021). This understanding has been underpinned by recent developments in strategies for analysing branched ubiquitin chains. One such approach engineered ubiquitin to include a TEV cleavage site after either Gly55 or Glu64, or both (Meyer and Rape, 2014). After substrate modification with these ubiquitin variants, subsequent incubation with TEV would collapse the modified forms of the substrate if the attached ubiquitin chains were branched. A second approach engineered a cell line in which an R54A ubiquitin mutant is expressed with simultaneous shRNA-based removal of endogenous ubiquitin. The R54A ubiquitin mutant removes a tryptic cleavage site, enabling quantification of unbranched K48 linkages, unbranched K63 linkages, and K48/K63 branched linkages by MS-based approached (Ub-AQUA-PRM). This approach, which may also utilise TUBEs for prior enrichment, provided evidence that heterotypic ubiquitin chain formation is dependent on collaboration between distinct E3 ubiquitin ligases, with K48/K63 chain production being mediated by TRAF6 and HUWE1, or ITCH and UBR5 (Ohtake et al., 2016; Ohtake et al., 2018).

Another breakthrough tool used to analyse heterotypic ubiquitin chain formation was the development of a bispecific antibody that detects K11/K48-linked chains (Figure 3D) (Yau et al., 2017). The bispecific antibody was generated from known sequences of K11- and K48-specific antibodies, using knobs-into-holes technology. After extensive validation steps, the authors used the bispecific antibody to analyse the products of the E3 anaphase promoting complex (APC/C) to confirm previous biochemical results demonstrating that it is capable of producing K11/K48-linked chains. The authors then used this bispecific antibody to identify a protein quality control pathway that functions in response to proteotoxic stress, via K11/K48-linked chain formation, with a range of factors identified, including BAG6, UBR5, HUWE1, and p97/VCP (Yau et al., 2017). In the future, production of such bispecific antibodies will be invaluable to help further understand the cellular function of other heterotypic ubiquitin types, such as K48/K63, especially in response to DNA damage, where it could be used to identify both substrates and regulators. Moreover, beyond bispecific antibodies that recognise heterotypic ubiquitin chain types, it may also be possible to generate bispecific antibodies that couple recognition of a specific ubiquitin chain type and recognition of the substrate itself, thereby generating an antibody that recognizes the ubiquitinated form of the substrate.

### Ub-AQUA, UbiCRest, UbiChEM and Ubi-clipping

To analyse ubiquitin chain types via bottom-up approaches, the ubiquitin-AQUA (absolute quantification of ubiquitin) method was developed to provide quantitative analysis of ubiquitin chain linkages by mass spectrometry (Kirkpatrick et al., 2006; Phu et al., 2011). This approach uses heavy labelled synthetic internal standard peptides to quantify the abundance of different ubiquitin tryptic peptides using selected reaction monitoring (SRM). More recently, multiple reaction monitoring (MRM) or parallel reaction monitoring (PRM) approaches can be used to improve the sensitivity of ubiquitin chain type detection (Ordureau et al., 2015). The Ub-AQUA method can be integrated into various proteomics workflows, including the prior enrichment of targets or chain types using TUBEs or linkage-specific antibodies. However, Ub-AQUA cannot be used to determine ubiquitin chain length, nor can it be used to quantify the abundance of heterotypic ubiquitin chains.

As an approach to overcome this obstacle, complex ubiquitin chain types can be analysed using linkage specific DUBs in an assay termed Ubiquitin Chain Restriction (UbiCRest) (Hospenthal et al., 2015). By using a panel of DUBs with known linkage specificities, ubiquitinated samples can be subjected to DUB assays and subsequent gel or immunoblot analysis using linkage specific antibodies to provide qualitative information on ubiquitin chain architecture (Figure 3E). Moreover, UbiCRest may also be used in conjunction with Ub-AQUA to quantitatively assess the linkage types in the products of the DUB reactions (Harris et al., 2021).

The application of middle-down mass spectrometry approaches in which ubiquitin is subject to restricted trypsin digestion under native conditions has proven applicable to detecting complex ubiquitin chain architectures (Valkevich et al., 2014; Ohtake et al., 2019). This method, termed ubiquitin chain enrichment middle-down mass spectrometry (UbiChEM-MS), is based on the observation that minimal trypsin digestion after position R74 liberates ubiquitin monomers with a GG motif attached at a lysine previously engaged in chain formation. As branching requires the addition of multiple ubiquitin subunits then minimal trypsin digestion will generate two or more GG motifs at lysine residues. Therefore, with chain branching, at least three distinct species would be observed by mass spectrometry, including mono-ubiquitin (Ub) at the ends of a chain, singly modified ubiquitin (^GG^Ub) within the linear chain, or doubly modified ubiquitin (^2xGG^Ub) at branch points. When this approach is combined with linkage-specific antibodies, the abundance of branching at the defined linkage can be defined. This approach was used to detect branching using a K11 specific antibody and demonstrated the formation of K11/K48 branches in response to proteasome and DUB inhibition (Rana et al., 2017).

In an alternative strategy to quantify branched ubiquitin chains, a method termed Ub-clipping was recently developed (Swatek et al., 2019). This method took advantage of the observation that the viral protease Lb^pro^ cleaves ubiquitin after R74, leaving ubiquitin with a GG remnant on a target substrate. More than one GG remnant indicates a branchpoint in the ubiquitin chain and can provide information on the polyubiquitin architectures by intact MS analysis (Figure 3F). To counter the effect of free unassembled monoubiquitin influencing the chain composition, a TUBE was used to remove monoubiquitin prior to Lb^pro^ treatment. This study quantified 10–20% of ubiquitin polymers existing as branched chains across 3 cell types, indicating that a substantial amount of branched ubiquitin can occur in cells.

In summary, there’s now a number of powerful MS-based tools that will provide the opportunity to identify and quantify changes in ubiquitin chain architecture at a much deeper mechanistic level. For the DDR, this promises to uncover novel components of ubiquitin signaling, site-specific changes in response to DNA damage, the dynamic changes of ubiquitin chain architecture, and how chain architecture promotes genome stability.

## Biochemical and Structural Approaches for Understanding the DNA Damage Response and Ubiquitin Signaling Mechanisms

A limiting factor for dissecting the precise mechanisms of ubiquitin signaling in DNA repair is the production of physiologically relevant, purified, and uniform components. In contrast, many of the key phosphorylation-dependent signaling components involved in DNA repair such as the PIKK-family kinases of ATM, ATR, and DNA-PK have been thoroughly investigated to reveal their mechanisms of action (Deshpande et al., 2017; Jansma et al., 2020; Chen S. et al., 2021; Chen X. et al., 2021; Chaplin et al., 2021; Hepburn et al., 2021; Tannous et al., 2021). Due to the complexity of ubiquitin signaling and the difficulty in producing specific and uniformly ubiquitinated proteins, there have been discrepancies in assigning the function of particular ubiquitin signals. In recent years, however, the development of techniques for *in vitro* biochemical reconstitution and the rapid expansion of methods for the structural investigation of these multi-factor assemblies has enabled greater consensus for the function of ubiquitin modifications in genome stability.

### Model *In Vitro* Systems

As described in the previous two sections, genetics, cell biology and cellular biochemistry have helped reveal the protein factors involved in the DDR, but the complexity of these systems limits the elucidation of mechanistic details of their activities and the pathways they are involved in. To circumvent this complexity, cell-free or reconstituted systems have been developed to enable greater control and design over the factors present and the types of signaling to occur.

**FIGURE 4:**
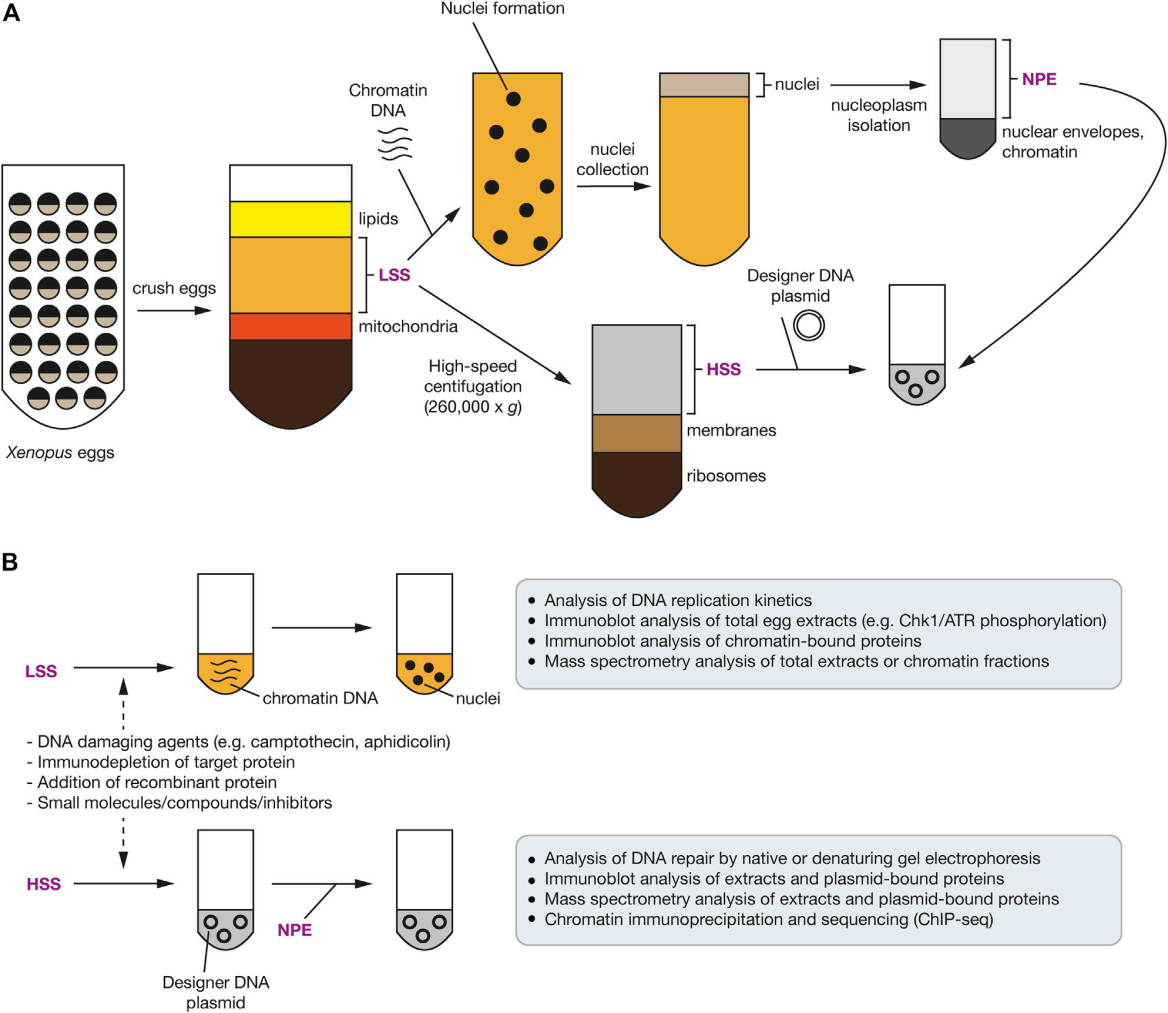
Use of *Xenopus* egg extracts to investigate the DDR and ubiquitin signaling. **(A)** Schematic representation of the common steps involved in preparing *Xenopus* egg extracts. Unfertilised eggs are crushed and a low-speed centrifugation of the crude extract produces cytoplasmic extract including membranes (LSS). High-speed centrifugation produces cytoplasmic extract without membranes or ribosomes (HSS). Incubation of the LSS with sperm chromatin, in the presence of ATP, induces nuclear envelope formation. Nucleoplasm (NPE) can be isolated by centrifugation after isolating these nuclei. **(B)** Upon addition of sperm chromatin DNA to the LSS, nuclei form and chromatin DNA undergoes replication. Addition of DNA damaging agents can be added to activate the DDR. Plasmid DNA with a defined site-specific DNA lesion, such as a replication barrier or ICL, can be added to the HSS, which is further supplemented with the NPE to stimulate DNA replication and repair of the site-specific DNA lesion. In both systems, a protein-of-interest can be immuno-depleted with antibodies and rescued using recombinant wild-type or mutant proteins.

*Xenopus* egg extracts have been used to study a variety of complex signaling pathways including DNA replication and termination, apoptosis, mitosis, and DNA repair mechanisms (Cupello et al., 2016; De Robertis and Gurdon, 2021; Gillespie et al., 2012; Hoogenboom et al., 2017; Willis et al., 2012). Many of the factors involved in mammalian DNA replication and repair are highly conserved in *Xenopus*, making this an excellent model system. Egg extracts from *Xenopus laevis* contain a high concentration of the factors required for proficient DNA replication and repair without needing the tailored production of all components, with addition of the low-speed supernatant (LSS) to demembranated sperm chromatin resulting in a complete round of DNA replication (Figure 4A). The requirement for membrane formation can be limiting in some cases, so alternatively, sequential addition of the high-speed supernatant (HSS) to DNA, such as plasmid DNA, followed by the highly concentrated nucleoplasmic egg extract (NPE) can trigger replication initiation in a synchronous manner. Such fine synchronisation and control over replication timing can be difficult in a cell culture setting. Furthermore, chemical perturbation of DNA replication and repair and ubiquitin signaling can be investigated with the treatment of egg extracts with compounds such as camptothecin (Topoisomerase I inhibitor) or aphidicolin (DNA polymerase *α* inhibitor) (Figure 4B). The control and reproducibility of this system allows experimental design with high spatial and temporal resolution, with typical assay outputs varying from immunoblot analysis of chromatin extracts over a particular time course, targeted enrichment of a particular protein of interest, or mass spectrometry for protein identification and/or analysis of PTMs in response to a particular DNA lesion (Gallina et al., 2021). Moreover, in recent years, this cell-free extract system has also been coupled with single-molecule techniques (Gruszka et al., 2020; Cameron and Yardimci, 2021). Lastly, to more accurately define the DDR and ubiquitin signaling in response to particular DNA lesions, specifically designed DNA plasmid templates can be used with the *Xenopus* egg extracts (Hoogenboom et al., 2017). For example, plasmids may be generated that contain a DNA-protein crosslink (DPC), interstrand crosslink or mimic a terminated DNA replication fork (Duxin et al., 2014; Deng et al., 2018; Larsen et al., 2019; Sparks et al., 2019).

Whilst the *Xenopus* egg extract system does allow a high degree of control over assay design, the ability to deplete a particular factor can be limiting, particularly as genetics approaches are not readily available, as noted above. Depletion from the egg extract requires the production of tailor-made antibodies to specifically immuno-deplete the protein of interest (POI) without targeting other factors involved in the same process as the POI. Moreover, rescue or add-back experiments require recombinant protein (wild-type and mutants) to be added at high concentrations, which can be an obstacle if such reagents cannot be produced or behave differently upon addition to egg extracts. For example, it was recently shown that the RPA complex-interacting E3 ligase RFWD3 ubiquitinates a range of substrates at stalled replication forks in *Xenopus* egg extracts (Gallina et al., 2021). However, difficulty in preparing active and specific recombinant RFWD3 has so far prohibited rescue experiments in this setting, whilst therefore also making reconstitution of this ubiquitin signaling *in vitro* a major challenge.

Beyond the *Xenopus* system, several groups have extended cell-free approaches by producing entirely reconstituted systems for specific cellular processes, with the reconstitution of DNA replication and some DNA repair events being notable major advances (Yeeles et al., 2015; Yeeles et al., 2017; Guilliam and Yeeles, 2020). Whilst this approach allows highly controlled experimental design, it requires that all the components of such a system are known. Thus, it can be difficult to fully reconstitute the dynamic DDR and ubiquitin signaling events found in a mammalian cell or *Xenopus* egg extract.

### Recombinant Tools for Investigating Ubiquitin Signaling

Cell-free systems provide a powerful biochemical alternative to more complex genetic and cell biology-based approaches. However, to obtain insights into the mechanistic functionality of DDR proteins and ubiquitin signaling, a more reductionist and purified system is required. Both prokaryotic (e.g., *E. coli*) and eukaryotic (e.g., yeast, insect and mammalian) expression systems have been used to produce recombinant proteins, with developments in multi-component co-expression, such as MultiBac, and endogenous tagging enabling larger protein assemblies to be purified with minimal steps and to high purity and yield (Bieniossek et al., 2012). This has been utilised for many signaling components in DDR, such as the PIKK-family kinases in conjunction with enzymatic assays, biophysical and structural techniques, and microscopy and single-molecule methods (Jansma and Hopfner, 2020).

**FIGURE 5:**
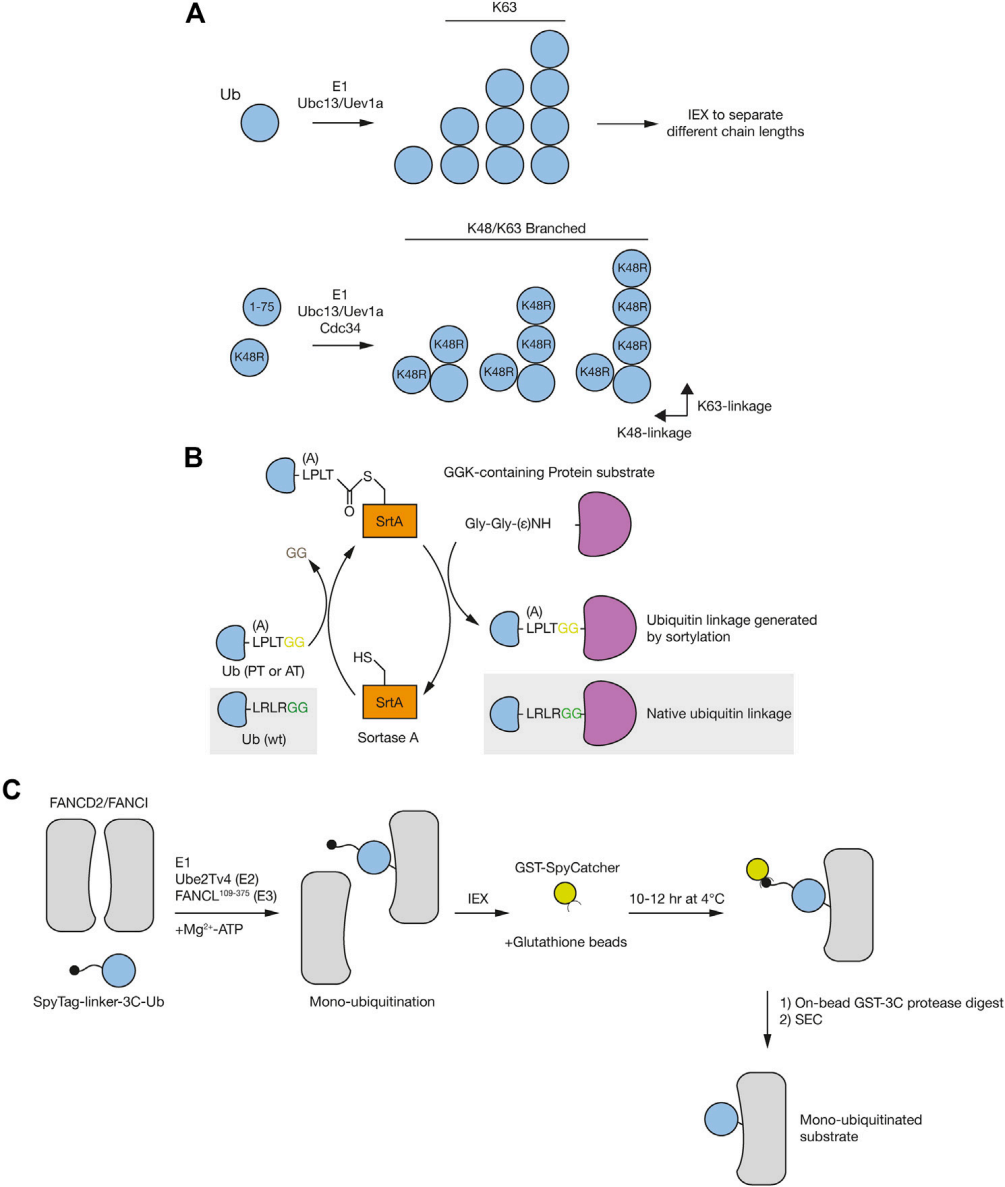
Preparation of designer substrates to investigate the DDR and ubiquitin signaling. **(A)** Using E2 conjugating enzymes and specific E3 ligases and/or DUBs enables the formation defined ubiquitin chain types. Ubiquitin chains of different lengths can be separated by ion-exchange chromatography (IEX). Use of specific ubiquitin mutants enables the formation of branched or more complex species. **(B)** A GGK-containing protein can be prepared by site-specific incorporation of the unnatural amino acid AzGGK using genetic-code expansion. *In vivo* Staudinger reduction converts AzGGK to GGK, which can undergo transpeptidation with a ubiquitin mutant containing a sortase recognition motif (LPLTG or LALTG) via SrtA. The resulting ubiquitinated protein displays a native isopeptide bond with R72P/R72A and R74T point mutations in the linker region. **(C)** Use of engineered E2 and E3 enzymes with tagged ubiquitin enables the efficient formation of specifically ubiquitinated substrates. Subsequent steps such as IEX, affinity purification and size-exclusion chromatography (SEC) enable enrichment of the uniformly ubiquitinated species.

An additional complication for ubiquitin signaling is the ability to produce uniformly ubiquitinated substrates in high yields, on the physiologically relevant ubiquitin modification site(s), and chain linkages and length. Generally, specific E3 ubiquitin ligases, and subsequent DUB treatment in some cases, can be used to produce ubiquitin chains of particular chain linkage types and lengths (Michel et al., 2018). Combining these scalable enzymatic methods with specific Lys-to-Arg or other mutations, such as in the hydrophobic patch or C-terminal Gly-Gly within ubiquitin, enables further control over the types, length and branching of ubiquitin chains (Figure 5A). Proteomic approaches such as Ub-AQUA and middle-down mass spectrometry can be used to validate the ubiquitin chain architecture produced (Ohtake et al., 2019). Furthermore, incorporating fluorophores and other functional chemical moieties into ubiquitin by semi-synthetic chemical or enzymatic methods, such as for activity-based probes described above, provides a chemical toolbox for creating a whole suite of ubiquitin-based substrates to investigate the activity of enzymes involved in ubiquitin signaling.

In order to investigate the specific function and mechanisms of ubiquitin signaling in DNA repair more bespoke methods for producing ubiquitinated substrates are required. For example, histone H2A ubiquitination can be produced by several different enzymes: BRCA1/BARD1 (K125/127/129), RNF168 (K13/15) and RING1A/B (K118/119) (Uckelmann and Sixma, 2017). Whilst the extent of ubiquitination can differ widely in these enzymatic-based preparations, the use of tagged-ubiquitin can be used to enrich for the modified form. Furthermore, depending on the preparation of the enzymes, the specificity of the enzyme or the target enzyme complex, the substrates may rarely be uniformly modified, particularly if neighbouring lysine residues can also be modified. In some cases, it has been possible to introduce the specific ubiquitinated site through the use of non-natural amino acids and semi-synthetic chemistry, however, the lack of a non-natural linkage (i.e., not an isopeptide bond) prevents cleavage by DUBs (Virdee et al., 2011). More recently, sortase-based approaches have enabled larger and more complex ubiquitinated proteins to be produced (Figure 5B) (Crowe et al., 2016; Fottner et al., 2019; Hofmann et al., 2020). These sortase-based methods allow the ability to produce ubiquitinated proteins both recombinantly and within a cellular environment. However, mutations near the C-terminus of ubiquitin mean the isopeptide linkage is not cleavable by DUBs, which is a substantial limitation when investigating such dynamic signaling events. Efforts to engineer sortase mutants to utilise the natural ubiquitin C-terminus are likely underway.

A recent notable example that demonstrates method developments to produce uniformly ubiquitinated species is the FANCI:FANCD2 complex. Described in more detail elsewhere, this heterodimeric complex is a key component in the ubiquitin-dependent Fanconi Anaemia pathway of DNA repair (Nalepa and Clapp, 2018). A critical junction in the FA pathway is the specific monoubiquitination of FANCD2 and FANCI. However, the function of this sequential multi-monoubiquitination was still unclear, hampered by an inability to produce the monoubiquitinated FANCI:FANCD2 complex at sufficiently high yields. As such, several groups have developed methods to efficiently produce mono-ubiquitinated FANCI:FANCD2 (Figure 5C). The Walden group developed a UBE2T variant that enables more efficient ubiquitin transfer to FANCD2 with FANCL alone, whilst maintaining target specificity (Chaugule et al., 2020). This was then combined with the use of the high affinity SpyTag/SpyCatcher system to purify the ubiquitinated species from other reaction constituents and unmodified substrate (Chaugule et al., 2019). Alternatively, the Deans group used an His-Avi-3C-tagged ubiquitin alongside known E2 and E3 enzymes for successful isolation of ubiquitinated species: UBE2T and FA core complex for the FANCI:FANCD2 complex, UBE2D3 (UbcH5c) for PCNA and BRCA1-BARD1 for H2A (Tan et al., 2020a). Thus, the continuing improvement in methods such as these will allow the uniform production of site-specific ubiquitinated substrates that will be key to understanding the mechanisms and function of ubiquitin modifications in the DDR. Whilst techniques to produce free ubiquitin chains are well established (Michel et al., 2018), methods to produce specifically ubiquitinated substrates relevant for DNA repair are still in their infancy. Progress has been made in some instances but more specialised systems and optimised protocols are likely required to be able to fully recapitulate some of these dynamic signaling pathways.

### Integrated Structural Techniques

**FIGURE 6:**
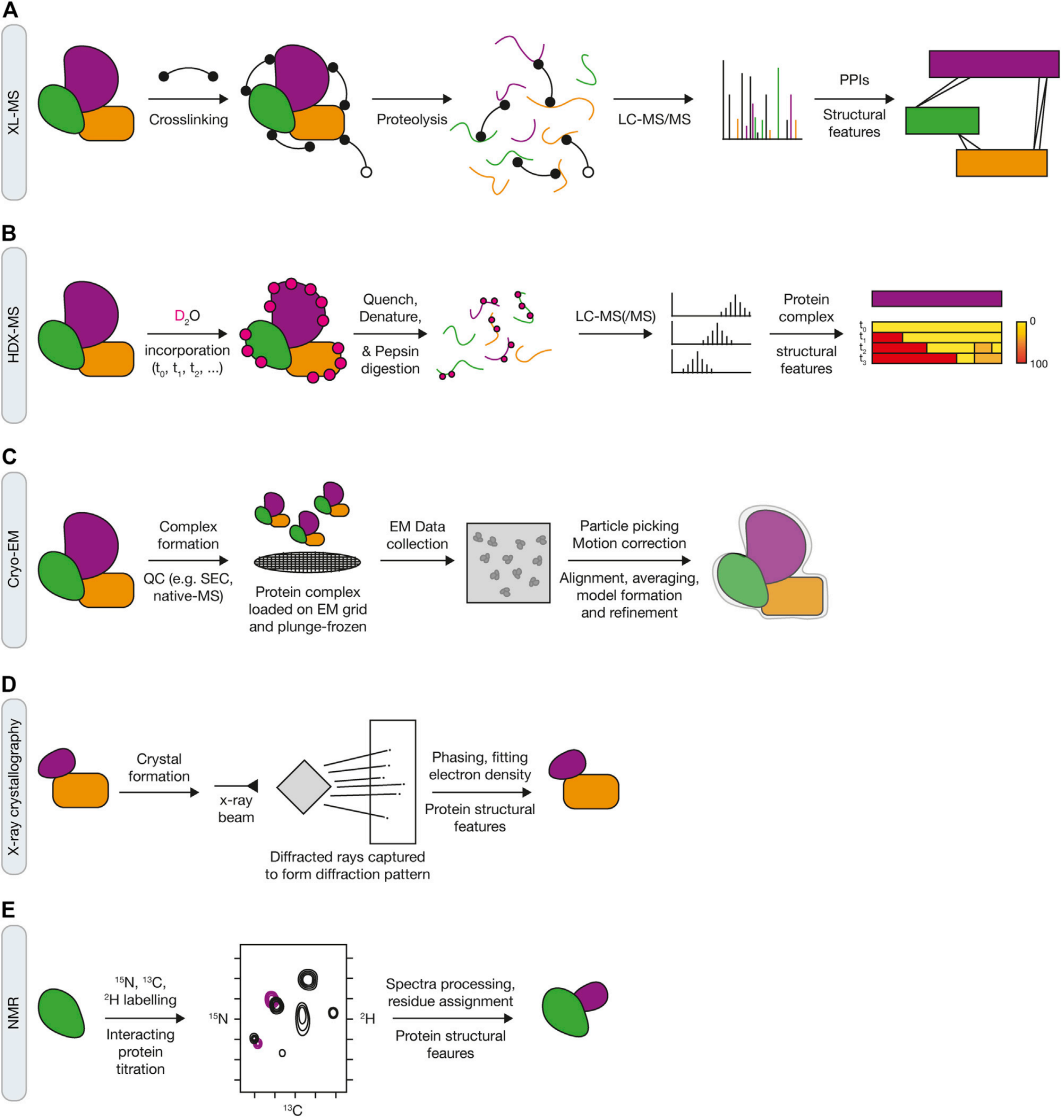
Integrated structural approaches to investigate mechanisms of ubiquitin signaling in the DDR. **(A)** Crosslinking with mass spectrometry (XL-MS) requires the use of chemical crosslinkers to react with amino acid side chains, such as BS3 NHS-ester chemistry for primary amines on lysine residues or N-termini of proteins. Subsequent proteolysis with Trypsin or LysC, tandem mass spectrometry and data analysis using specialised software enables identification of crosslinked peptide species. **(B)** Hydrogen-deuterium mass spectrometry (HDX-MS) relies on the incorporation of ^2^H into the protein by incubation in deuterated water. The experiment uses a time course of ^2^H-incorporation followed by a rapid quenching and denaturation step at pH 2.5 before pepsin digestion and mass spectrometry analysis. Specialised data analysis pipelines can assess differences in ^2^H-incorporation for a protein across experimental conditions. **(C)** Single particle cryo-electron microscopy (cryo-EM) has recently evolved as a technique to get high-resolution structural data of larger multi-protein assemblies. Protein samples go through several stages of quality control (QC) via biochemical and biophysical techniques (e.g., SEC and native mass spectrometry) before loading onto carbon-coated EM grids, plunge-freezing in liquid ethane and data collection using high-power electron microscopes. **(D)** X-ray crystallography and **(E)** nuclear magnetic resonance (NMR) are established techniques to gain atomic-level resolution of protein structures that relies on the formation of crystals and isotopically labelled proteins, respectively.

Probably the greatest technical advancement for investigating the mechanisms of ubiquitin signaling in DNA repair is the development of high resolution, single particle cryo-electron microscopy (Cryo-EM, Figure 6) (D'Imprima and Kuhlbrandt, 2021; Glaeser, 2019; Kim et al., 2018). The enhancements in microscope design, detection methods and rapid software development has enabled high resolution structures of protein assemblies to be solved that would not have been thought possible little over a decade ago (Scheres, 2012; Fernandez-Leiro and Scheres, 2017; Punjani et al., 2017; Zivanov et al., 2018). Structures have now been solved of large E3 ligases and DUBs with and without their substrates (Rabl et al., 2019; Shakeel et al., 2019; Wang et al., 2021; Witus et al., 2021). Combining these novel structures with further biochemical, proteomic, and biophysical approaches has led to a new era of integrated structural approaches, whereby this structural information can be corroborated with genetics and cell-based approaches.

Recent structural studies of the factors involved in FA pathways, such as the FA core complex, the FANCI:FANCD2 heterodimer and the USP1-UAF1 DUB complex, are prime examples (Li et al., 2020). The ability to produce uniform ubiquitinated substrates in combination with state-of-the-art cryo-EM and mass spectrometry techniques has provided a much deeper insight into the mechanisms of ubiquitin signaling DDR pathway. Improvements in multi-subunit co-expression enabled the FA core complex, the E3 ligase responsible for FANCI:FANCD2 complex monoubiquitination, to be purified in high yields for structural investigation (Shakeel et al., 2019; Wang et al., 2021). In addition to using the described improvements in cryo-EM, native mass spectrometry was used to analyse subunit stoichiometry and complex uniformity. This level of sample quality assurance in conjunction with advanced structural methods provides important information about the protein complex and aids in forming conclusions about the functional significance of solved structures. Further mass spectrometry-based methods, including crosslinking with mass spectrometry (XL-MS, Figure 6A) and hydrogen-deuterium exchange mass spectrometry (HDX-MS, Figure 6B), also proved invaluable in helping to assign subunit and domain locations within such a large and complex assembly. Indeed, the rise in quality and use of single particle cryo-EM has occurred alongside technological advances in biological and structural mass spectrometry (Chen and Rappsilber, 2019; Mistarz et al., 2016; O'Reilly and Rappsilber, 2018; Walzthoeni et al., 2013). Mass spectrometers are becoming increasingly sensitive and the depth of sequence coverage for proteomics experiments has improved several-fold. This, along with developments in software packages and data analysis pipelines, has vastly enhanced the extraction of robust structural proteomics data and opened up wider access to these types of methods. Despite the advancement in technology for these structural mass spectrometry methods however, there have been inconsistencies in analysing and interpreting the resulting data and as a result, there has been a move to produce a standardised set of parameters in experimental design (Iacobucci et al., 2019; Masson et al., 2019; Leitner et al., 2020).

Improved sample preparation and advances in cryo-EM also proved fruitful in assigning the function of the sequential monoubiquitination of the FANCI:FANCD2 complex. Cryo-EM reconstructions, alongside XL-MS and DNA-binding experiments, suggested a role for the monoubiquitination in transforming FANCI:FANCD2 into a DNA clamp (Alcon et al., 2020; Tan et al., 2020b; Wang R. et al., 2020). In addition to this novel finding, the ubiquitin of one protomer (i.e., FANCD2 or FANCI), binds to the other protomer within the complex, effectively shielding it from a potential role in the recruitment of other DNA repair factors via their ubiquitin binding domains. This clamp role for the ubiquitinated FANCI-D2 has been proposed to protect the underlying DNA during repair of the lesion, with the removal of the modification enabling FANCI:FANCD2 to be released from the site upon repair. Furthermore, the mechanism for the removal of ubiquitin from FANCD2 was also clarified by cryo-EM and crystallography experiments with USP1-UAF1 (Rennie et al., 2021). Crystals of the apo- and ubiquitin-bound form of USP1-UAF1, in conjunction with cryo-EM reconstructions of the enzyme-substrate complex, revealed important details of the specificity and regulation of this reaction. Amino acid residues at the FANCI-UAF1 interface, including those of known ATR phosphorylation sites, were shown to be critical for regulating USP1-mediated removal of the FANCD2-Ub mark, corroborating previous genetic and biochemical data (Tan et al., 2020c). Collectively, these findings show that the recent technical developments in structural biology have led to fundamentally important discoveries of how ubiquitin signaling regulates the DDR.

### Recapitulating the DNA Damage Response and Ubiquitin Signaling in a Chromatin Context

A prominent question in the DDR field is how ubiquitin signaling events occur in the context of chromatin. The production of recombinant nucleosomes for investigating chromatin-based signaling mechanisms has been demonstrated within the epigenetics field (Luger et al., 1997; Dyer et al., 2004; Dao et al., 2020; Liu et al., 2020). Furthermore, preparing ubiquitinated nucleosome core particles, similar to the enzymatic and chemical methods noted above, has become increasingly common to investigate the regulatory mechanisms in chromatin processes, including how DDR factors function in the context of chromatinised DNA lesions (McGinty et al., 2014; Nguyen et al., 2014; Worden et al., 2019; Worden et al., 2020). For example, the critical choice between HR and NHEJ has been investigated with structural investigations of how 53BP1 interacts with nucleosomes containing H4K20me2 and H2AK13/15-Ub via its Tandem Tudor domain and ubiquitin-dependent recruitment motif (UDR), respectively (Wilson et al., 2016). Furthermore, a recent cryo-EM structure of BRCA1/BARD1 with UBE2D3 (UbcH5c) on a nucleosome also provided mechanistic details for the specificity of the enzyme for H2AK125/127/129 ubiquitination (Witus et al., 2021). Thus, developments in cryo-EM, such as phase plates and sample preparation, paves the way for further ubiquitin-modified nucleosome-bound complexes to be solved in the context of the DDR (Chua et al., 2016; Chua and Sandin, 2017).

Whilst mono- or di-nucleosome containing structures have been solved, how DNA repair factors and ubiquitin signaling events function in the context of higher order chromatin is still relatively unclear. *In vitro* assembly and subsequent structural reconstruction of chromatin relies on forming unnaturally rigid nucleosome arrays to reduce sample heterogeneity. This is added to the complication of including the relevant PTMs at the correct sites and including all the necessary protein factors within the DNA repair machinery. With increasing capabilities in reagent production and data acquisition by cryo-EM, it might be possible to reconstitute some of these complex ubiquitin signaling pathways and visualise them by time-resolved techniques. However, it is unlikely that the precise dynamics of these reactions in a chromatin context can be recapitulated by structural techniques noted here alone and perhaps single molecule techniques can help to fill these mechanistic gaps alongside other experimental systems.

## Future Perspectives

The development and application of the myriad methods and tools discussed here have helped shape our understanding of how ubiquitin signaling regulates the DDR over the past few years. Whilst the vast complexities of ubiquitin signaling are now starting to be decoded, future work will require integrated multidisciplinary approaches to gain a deeper mechanistic understanding of these processes both *in vitro* and in *in vivo*, with genetics, proteomics and biochemical methods critical to the success of this. Understanding the limitations of these technologies will also lead to innovation and the creation of new tools that can be applied to the DDR.

For genetics, CRISPR-Cas9 has revolutionised biological science over the past decade. This technology has converged with the recent explosion in small molecules that have been designed to target the DDR and UPS, and their associated pathologies. The intersection of these two advances has facilitated transformational gene discovery within the DDR and UPS, uncovered novel sensitisation and resistance mechanisms and revealed new SL interactions. Further genome editing capabilities will continue to drive this progress, such as base editing screens (Cuella-Martin et al., 2021; Hanna et al., 2021). Moreover, whilst most of these screening approaches have been used in forward modalities, reverse genetics screens are coming to the fore with advances in both arrayed and pooled sgRNA libraries for image-based approaches (Feldman et al., 2019; Askary et al., 2020; Wheeler et al., 2020; Chandrasekaran et al., 2021; Kanfer et al., 2021; Lawson and Elf, 2021; Yan et al., 2021).

For proteomic approaches, the past decade has also seen the development of many novel techniques coupled with advances in MS approaches to map proteins at the replication fork and identify ubiquitinated proteins. The role of ubiquitination at the replication fork and its function in the DDR remains far from complete. The identification of novel ubiquitin-regulating enzymes and factors continues to expand and further our understanding of ubiquitin-mediated signaling. Furthermore, approaches that better interrogate ubiquitin chain architecture on proteins will help to provide insight into the extent of mixed and branched chain types and their role in the DDR. An integrated approach that utilises multiple proteomic methods, including those described here, may help to assign function to these chain types and identify how they are regulated. While not discussed in detail in this review, the interplay between ubiquitin, UBLs, and post-translational modifications provides an additional level of regulation that contributes to the complexity of the ubiquitin code and must also be considered. Further technical developments in MS data acquisition enabling greater detection and profiling of ubiquitin modifications across multiple samples in parallel may also help to achieve better resolution and corroborate findings from large-scale DNA damage screens.

Developments in cryo-EM, structural mass spectrometry, and recombinant tool development described here build on the plethora of data available via tailored biochemical and biophysical data, X-ray crystallography (Figure 6D), and NMR approaches (Figure 6E). It is becoming increasingly clear that to understand the mechanisms of how ubiquitin modifications function in DNA repair, highly specific reagents and multiple integrated experimental systems need to be utilised. Moreover, although not discussed in detail here, future directions in single molecule techniques and super-resolution microscopy will allow greater resolution of some of these signaling machines within the context of a cellular environment. Already, temporal resolution of protein signaling can be resolved *in vitro* using cryo-EM (Miller et al., 2019). Thus, perhaps we are not too far away from obtaining high spatial and temporal resolution for DDR and ubiquitin-dependent signaling events in real-time.

Collectively, in light of recent technological advances, as well as novel insights from a variety of disciplines, it is conceivable that we are on the precipice of unravelling the complexity of ubiquitin signaling mechanisms in DNA repair through interdisciplinary approaches at an unprecedented level.
